# Evolutionary legacy of a forest plantation tree species (*Pinus armandii*): Implications for widespread afforestation

**DOI:** 10.1111/eva.13064

**Published:** 2020-07-27

**Authors:** Yun Jia, Richard I. Milne, Juan Zhu, Lian‐Ming Gao, Guang‐Fu Zhu, Gui‐Fang Zhao, Jie Liu, Zhong‐Hu Li

**Affiliations:** ^1^ Key Laboratory of Resource Biology and Biotechnology in Western China Ministry of Education College of Life Sciences Northwest University Xi’an China; ^2^ CAS Key Laboratory for Plant Diversity and Biogeography of East Asia Kunming Institute of Botany Chinese Academy of Sciences Kunming Yunnan China; ^3^ Institute of Molecular Plant Sciences School of Biological Sciences University of Edinburgh Edinburgh UK; ^4^ Germplasm Bank of Wild Species Kunming Institute of Botany Chinese Academy of Sciences Kunming Yunnan China

**Keywords:** afforestation, forest plantations, niche divergence, *Pinus armandii*, spatial genetic structure

## Abstract

Many natural systems are subject to profound and persistent anthropogenic influence. Human‐induced gene movement through afforestation and the selective transportation of genotypes might enhance the potential for intraspecific hybridization, which could lead to outbreeding depression. However, the evolutionary legacy of afforestation on the spatial genetic structure of forest tree species has barely been investigated. To do this properly, the effects of anthropogenic and natural processes must be examined simultaneously. A multidisciplinary approach, integrating phylogeography, population genetics, species distribution modeling, and niche divergence would permit evaluation of potential anthropogenic impacts, such as mass planting near‐native material. Here, these approaches were applied to *Pinus armandii*, a Chinese endemic coniferous tree species, that has been mass planted across its native range. Population genetic analyses showed that natural populations of *P. armandii* comprised three lineages that diverged around the late Miocene, during a period of massive uplifts of the Hengduan Mountains, and intensification of Asian Summer Monsoon. Only limited gene flow was detected between lineages, indicating that each largely maintained is genetic integrity. Moreover, most or all planted populations were found to have been sourced within the same region, minimizing disruption of large‐scale spatial genetic structure within *P. armandii*. This might be because each of the three lineages had a distinct climatic niche, according to ecological niche modeling and niche divergence tests. The current study provides empirical genetic and ecological evidence for the site‐species matching principle in forestry and will be useful to manage restoration efforts by identifying suitable areas and climates for introducing and planting new forests. Our results also highlight the urgent need to evaluate the genetic impacts of large‐scale afforestation in other native tree species.

## 1 INTRODUCTION

The rate of deforestation is rapidly increasing all over the world (Hansen et al., 2013) and will result in unprecedented biodiversity losses (Barlow et al., 2016) and consequent reduction of ecosystem functions. As a potential solution, afforestation has been widely adopted by many countries around the world, for climate change mitigation, protection of natural forests, replacement of lost tree cover, facilitation of natural regeneration, and provision of forest products (Bastin et al., 2019; Canadell & Raupach, 2008; Carnevale & Montagnini, 2002). The scale of afforestation has rapidly increased in the past decades, with plantations covering 277.9 million ha, and accounting for 6.95% of global forest area by 2015 (Payn et al., 2015). China has been leading global afforestation efforts, with 78.9 million ha of planted forest area, which is 28% of the global total and by far the most in the world (Payn et al., 2015). This extensive afforestation was supported by several Key Forestry Programs (SFAPRC, 2014). In particular, the Grain for Green Project (GFGP) had re‐established 28.20 million ha of forest in 25 of China’s 31 mainland provinces by 2013 (SFAPRC, 2014), making it currently the largest revegetation program conducted anywhere (Hua et al., 2016).

The ecological impacts of large‐scale afforestation have attracted much attention (Peng et al., 2014). One issue is lack of species diversity in plantings (Hua et al., 2016); for example, in China, most forests planted are monocultures, and just ten species (e.g., *Cunninghamia lanceolata*, *Larix gmelinii*, *Pinus massoniana*, *P. tabuleaformis*, *Cupressus funebris*) account for 73% of total plantation area (SFAPRC, 2014). Planting any exotic species entails a small but significant risk that it might become invasive, if they are able to invade habitats and attain higher fitness than native species (Knowler & Barbier, 2005; Schutzenhofer, Valone, & Knight, 2009). This can be avoided by planting within a species’ native range, but that creates another concern that has received far less attention: the genetic effects of large‐scale plantings on native populations (Laikre, Schwartz, Waples, & Ryman, 2010). If introduced genotypes or alleles have a fitness advantage and/or they outnumber natives, the natural populations may be threatened by genetic swamping (Anttila, King, Ferris, Ayres, & Strong, 2000; Hufford & Mazer, 2003), or if the advantage is large, simple replacement by a more competitive genotype (Bayms, 2008). Furthermore, gene flow into native populations, especially if all plantings come from a common source, could lead to genetic homogenization across large parts of the planted species’ natural range (Olden, Poff, Douglas, Douglas, & Fausch, 2004). Nonlocal exotic plantations have significantly affected the genetic composition of the offspring of nearby conspecific populations in several cases (Unger, Heuertz, Vendramin, & Robledo‐Arnuncio, 2016), with proportions of introgressed offspring exceeding 40% in some cases (Steinitz, Robledo‐Arnuncio, & Nathan, 2012). However, the long‐term genetic consequences of large‐scale plantings, across the full range of a species, have not yet been properly investigated (Steinitz et al., 2012). The first stage of any such investigation would be to determine whether planted material is sourced from other regions. If that is the case, it would facilitate between‐region gene flow that would not otherwise happen naturally.

Examining the full effect of human‐induced intraspecific gene flow requires examination of spatial–temporal genetic variation across a species’ range (Avise, 2000) and is best conducted in concert with examination of naturally occurring genetic variation. Historical events such as orogenies and monsoon development, and climatic oscillations such as glacial cycles, affect geographic distribution of genetic variation among populations (Antonelli et al., 2018; Avise, 2000). Sometimes allopatric divergence and speciation may ensue (Avise, 2000; Li et al., 2013), and speciation events are commonly associated with adaptation to different local or regional environments (Maria et al., 2015). For dominant forest trees, large effective population sizes and long generation times can influence and obscure the genetic and phenotypic signals left during the diversification process (Li et al., 2013; Steinitz et al., 2012). Where large areas have been afforested, as noted above for China, this will complicate the signature of past biogeographic events and vice versa; hence, to fully examine any of these processes, all should be studied together (Ortego, Noguerales, Gugger, & Sork, 2015).

*Pinus armandii* is an evergreen montane coniferous tree species, whose natural range is concentrated in central and southwestern China (Farjon & Filer, 2013; Ma, 1989). Parts of its range have a dynamic geo‐climatic history (Clift & Webb, 2019; Wang et al., 2012), leading to a complex biogeographic history, with extreme environmental heterogeneity and diverse historical components that have helped shape one of the richest floras on the planet (Qian & Ricklefs, 2000). The species has a long history of felling for timber and industrial raw materials (Ma, 1992), leading to widespread deforestation and even serious ecological degradation in southwest China. To alleviate this phenomenon and protect regional water resources, the Chinese government has implemented large‐scale afforestation using *P. armandii* since the late 1950s (Ma, 1992), although success rates before 1980 were limited (Ma, 1992; Wang & Hong, 2004). A total of area 162,573 ha was planted with *P. armandii* between 1999 and 2010 under the GFGP, especially in the barren mountains of southwestern China (Yao et al., 2014), using a mixture of aerial seeding, artificial seeding, and seedling transplantation. Since 1980, provenance studies have been used to improve the adaptability and survival rate of planted material in China (Ma, 1989, 1992), but little is known about how this rapid anthropogenic increase in its numbers has affected the range‐wide genetic structure of *P. armandii*.

This rapid mass afforestation, often placing planted trees in the vicinity of native populations, forms a large‐scale experiment that provides an opportunity for studying the risk of genetic homogenization and other potential effects of planted to native gene flow within this species. Previous molecular analyses detected clear genetic divergence between northern and southern populations of *P. armandii* (Liu et al., 2014; Liu, Jin, Wei, & Wang, 2019), but beyond this, the details of spatial–temporal intraspecific differentiation remain unclear, and the genetic effects of afforestation are therefore unknown. Thus, *P. armandii* represents an excellent study system with which to simultaneously examine the effects of both natural historical events and recent anthropogenic mass afforestation on a major forest tree species.

In this study, we sought to tease apart the evolutionary legacy of *P. armandii* from that of mass planting, by contrasting phylogeographical signals from natural and planted populations. To this end, variation was examined across multiple microsatellite markers, plus chloroplast, mitochondrial, and nuclear DNA fragments, backed up by full chloroplast genome sequencing of selected individuals. We analyzed the patterns of genetic differentiation within *P. armandii* and employed a robust niche dynamics framework to compare climatic niche between intraspecific lineages. From this, we inferred divergence pathways, tested for environmental niche differentiation, and assessed the effects of forest plantations on the genetic composition of *P. armandii* natural populations. The results will serve as an important basis for future genetic monitoring in large‐scale afforestation initiatives and as a case study for the effect of mass afforestation within a species’ native range.

## 2 MATERIALS AND METHODS

### 2.1 Sampling area and plant material

**Figure 1:**
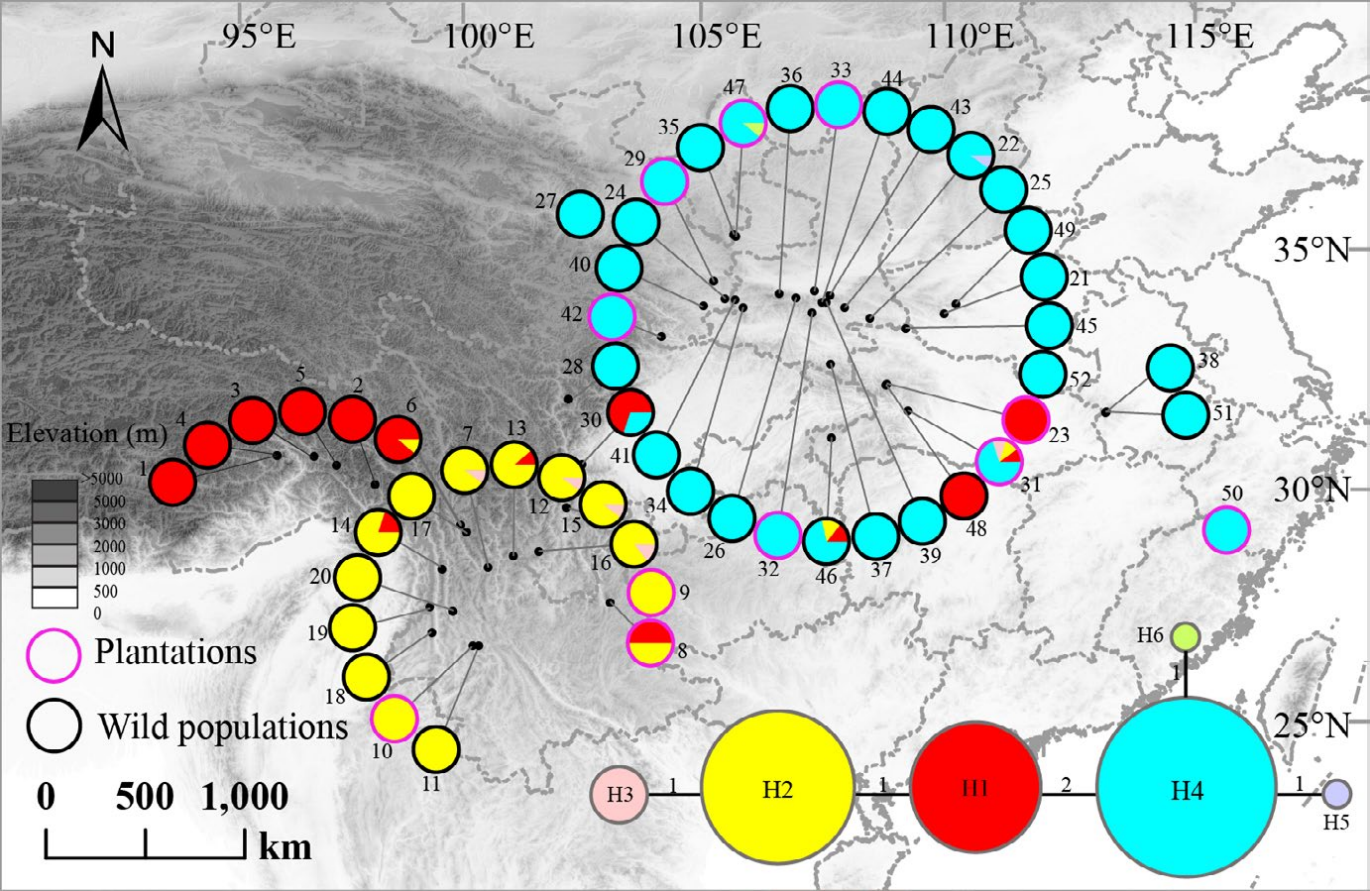
Geographic distribution and network of the chloroplast (cp) DNA haplotypes (H1‐H6) detected in *P. armandii*. The purple and black circles represent plantation and wild populations, respectively

Material of *P. armandii* was sampled from 41 natural populations from the species’ full range across central and southwestern China, plus 11 planted populations from Yunnan, Shaanxi, Gansu, Hubei, and Zhejiang provinces (Figure 1; Table S1). Because plantings before 1980 were largely unsuccessful (Wang & Hong, 2004), all planted populations we sampled were planted in the 1980s, on sites that were previously barren or farmland with no *P. armandii* present, according to local information and references (see Table S1). From all of these, a total of 696 mature trees were sampled that were between 25 and 90 years old, taking pine needles from each for DNA extraction and further molecular analysis (Figure 1; Table S1), that is, DNA sequencing, microsatellite genotyping, and chloroplast genome sequencing.

**Figure 2:**
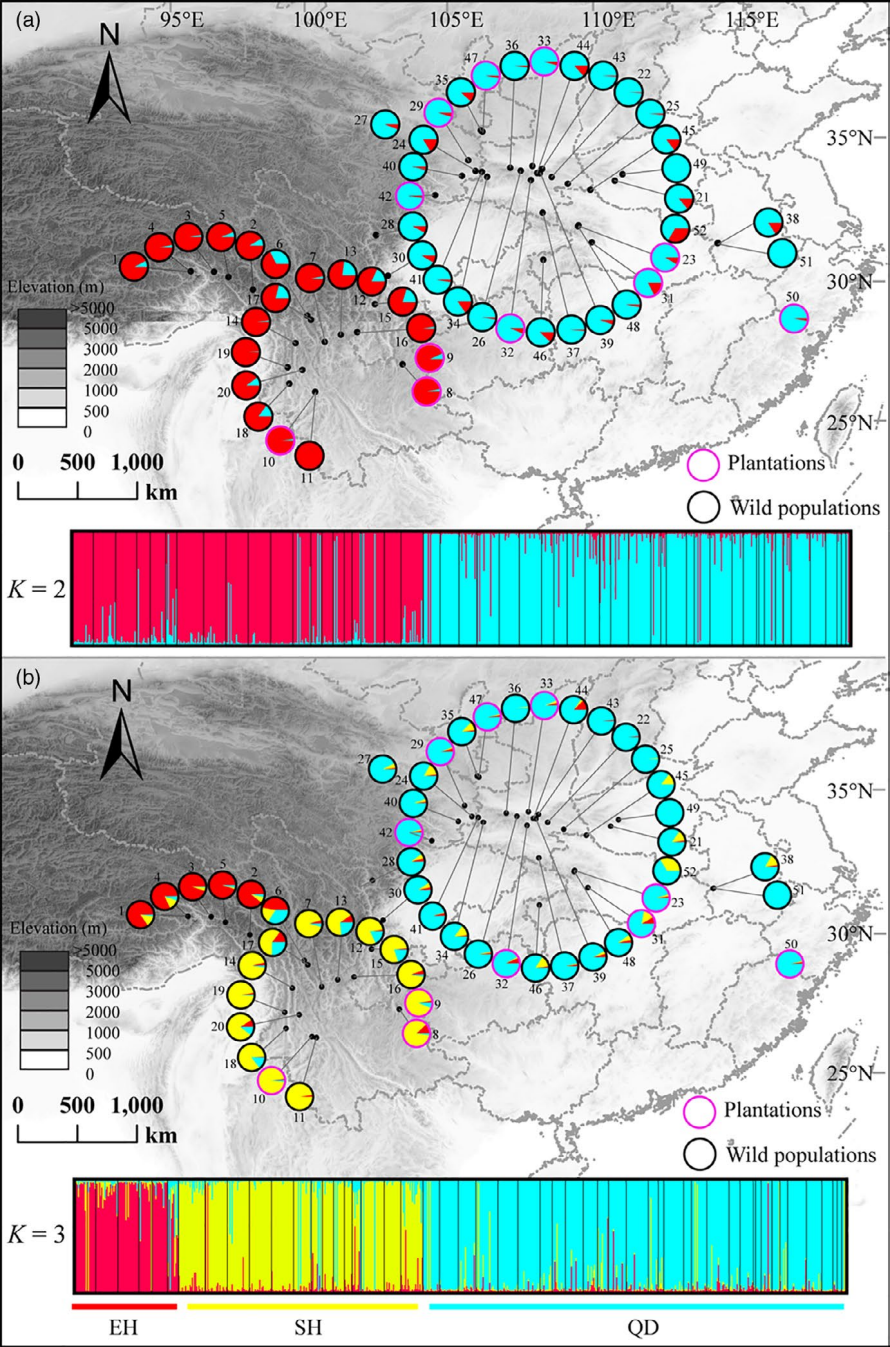
Structure analysis results and resultant map of genetic composition of each population in *P. armandii*. The *K* = 2 (a) and *K* = 3 (b) clusters are shown. For each *K* value, results of the run with the highest value of LnPD were used. The purple and black circles represent plantation and wild populations, respectively

Total genomic DNA was isolated using plant genomic DNA kits (Tiangen, Beijing, China). The genetic variation and structure of *P. armandii* were estimated based on a large‐scale population genetic dataset, comprising markers with both biparental and uniparental inheritance modes. Using twelve pairs of microsatellite primers, twelve nSSR regions were successfully amplified from all 696 sampled individuals (Table S2). Forward microsatellite primers were 5′‐end fluorescently labeled using either FAM or TAMRA (Applied Biosystems). PCR fragments were separated on an ABI 3730 xl DNA Sequencer and individually assessed using GENEMAPPER v4.0 (Applied Biosystems). In addition, following the protocols of Jia et al. (2018), one chloroplast (cpDNA) fragment, *ycf1*, and two nuclear fragments, *AGP*6 and *LFY*, were successfully amplified and sequenced for 466, 201, and 44 individuals, respectively. Following on from STRUCTURE results that separated out three geographic lineages (Figure 2), we selected between two and five individuals from each lineage, making 12 in total, for chloroplast genome sequencing; these together covered the full geographic range of *P. armandii* (Table S3). Additionally, sequences from two mitochondrial DNA (mtDNA) fragments, *nad5* intron 1 and *nad7* intron 1, were obtained for 193 individuals, from Li et al. (2015). Therefore, we were able to examine variation in *P. armandii* based on markers from three different genomes and hence with different inheritance modes.

### 2.2 DNA sequence analysis

All DNA sequences were visually inspected, edited, and aligned using MEGA v6 (Tamura, Stecher, Peterson, Filipski, & Kumar, 2013). For methodological details of DNA sequences analyses, see Appendix 1.

Divergence time was estimated with BEAST v1.7.5 (Drummond, Suchard, Xie, & Rambaut, 2012), using 12 published *Pinus* chloroplast genomes together with 12 newly sequenced individuals of *P. armandii* (Table S3). Due to the lack of credible fossils from *P. armandii*, we applied a crown node age of *Pinus*, that is, the divergence of the two subgenera *Pinus* and *Strobus*, of 85 million years. This followed Willyard, Syring, Gernandt, Liston, and Cronn (2007), who applied this date based on silicified fossil wood of subgenus *Strobus* from Late Cretaceous (85.8–83.5 Mya; Meijer, 2000). Convergence was checked using TRACER v1.5 (Rambaut & Drummond, 2009).

### 2.3 Microsatellite data analysis

Hardy–Weinberg equilibrium and linkage disequilibrium were tested for using FSTAT v2.9.3 (Goudet, 2001). In addition, MICROCHECKER v2.2.3 (Van Oosterhout, Hutchinson, Wills, & Shipley, 2004) was used to check the presence of null alleles. A total of twelve pairs of microsatellite markers were used for subsequent analysis. Genetic diversity parameters were computed using GENALEX v6.5 software (Peakall & Smouse, 2006). Significant differences between wild and planted populations were quantified using Kruskal–Wallis tests. We also performed AMOVA with all native *P. armandii* samples pooled, and three specific analyses considering separately each of the three regional groups that were identified for the analysis in ARLEQUIN v3.5 (Excoffier & Lischer, 2010). Pairwise *F*_ST_ was used to assess population differentiation within and among regions and populations (Excoffier, Smouse, & Quattro, 1992).

The Bayesian model‐based clustering software STRUCTURE v2.2.3 (Pritchard, Stephens, & Donnelly, 2000) was used to infer distinct gene pools using combined microsatellite, *AGP*6 and *LFY* data, for wild populations alone, planted material alone, and the full dataset. A rarefaction microsatellite dataset which include same number of individuals as nuclear gene dataset was also analyzed in STRUCTURE. The analyses were run using the admixture model with correlated allele frequencies. The optimal number of clusters was determined by calculating DeltaK (*ΔK*) (Evanno, Regnaut, & Goudet, 2005) using STRUCTURE HARVESTER (Earl & VonHoldt, 2012).

### 2.4 Genetic migration analyses

In order to examine historical genetic migration between regions, based on the microsatellite datasets, we used the coalescent‐based program MIGRATE‐N v3.6 (Beerli, 2006) to generate pairwise estimates of migration rates (*N*m) between the three identified regional groups. To assess patterns of recent migration, we also estimated interpopulation migration rates (within 2–3 generations) using a Bayesian approach in BAYESASS v3.0 (Wilson & Rannala, 2003).

### 2.5 Lineage divergence and demographic history

**Figure 3:**
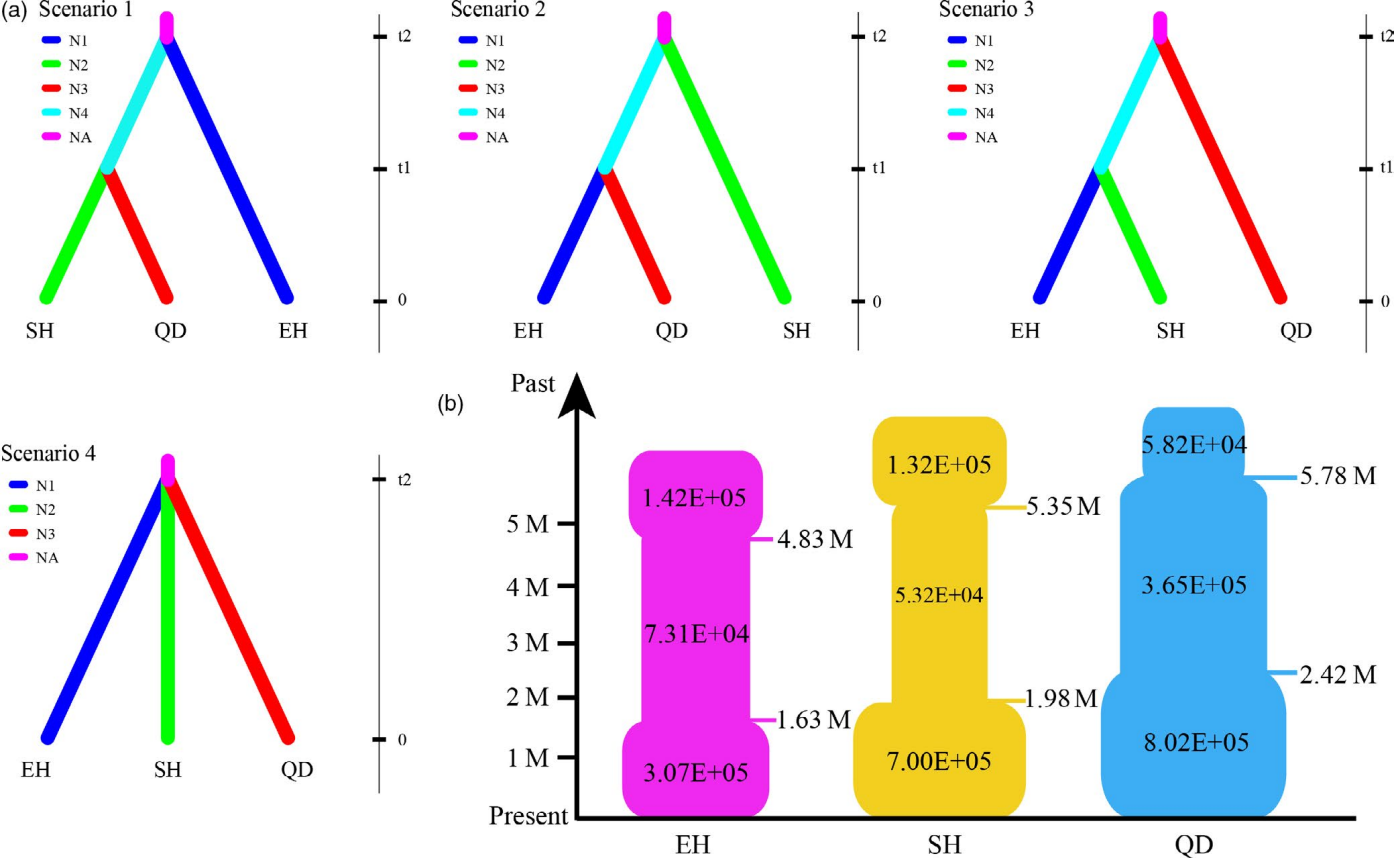
(a) The four scenarios for the population history of the three lineages (EH, SH, and QD) in DIYABC. (b) Schematic representation of four demographic models of changes in population size tested within the three lineages (EH, SH, and QD) in *P. armandii*

**Figure 4:**
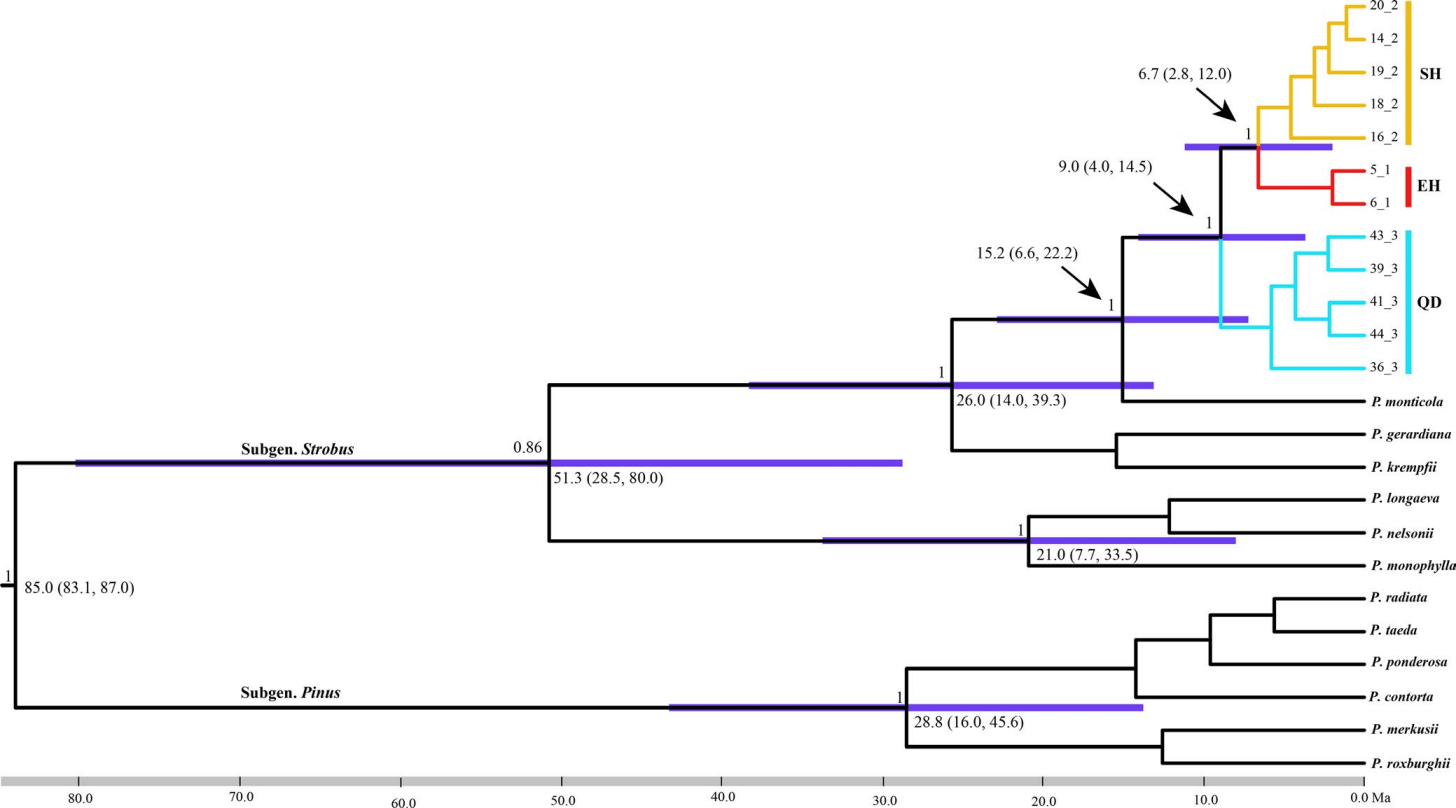
Phylogenetic relationships and divergence times of *P. armandii* based on BEAST analysis. Blue bars and the numbers below the bars indicate 95% highest posterior densities of divergence times (Ma). Posterior probabilities are labeled on each node. Red, yellow, and blue branches represent EH, SH, and QD lineages, respectively

To estimate plausible scenarios of divergence and population dynamics within *P. armandii*, approximate Bayesian computation (ABC) was performed in DIYABC v2.04 (Cornuet et al., 2014). This analysis treated as separate three regional subgroups or lineages (East Himalaya: EH, South Hengduan Mountains: SH, and Qinling‐Daba Mountains: QD) that were clearly identified based on STRUCTURE and phylogenetic results (Figures 2, 4). Four historical population divergence scenarios for these lineages were compared by DIYABC analysis: scenarios 1, 2, and 3 differed in that the first diverging lineage was EH, SH, or QD, respectively, whereas under scenario 4, the three lineages diverged simultaneously. We assumed uniform priors on all parameters and used a goodness‐of‐fit test to check the priors of all parameters before implementing the simulations (Figure 3; Table S4). To estimate the divergence times among the three lineages, the average generation time of *P. armandii* was assumed to be 25 years, following Ma, Szmidt, and Wang (2006).

In addition, DIYABC was used to simulate and examine population demographic changes in the recent past. We separately tested the following four scenarios of demographic changes for each of the three lineages: continuous shrinkage, continuous expansion, shrinkage–expansion, and expansion–shrinkage. DIYABC allows selection of the demographic scenario that best fits the data and parameters of interest (Cornuet et al., 2014).

### 2.6 Ecological niche modeling

Only wild occurrences have been taken into account for this and all subsequent analyses. Ecological niche modeling (ENM) analyses were performed with MAXENT v3.3.3k (Phillips, Anderson, & Schapire, 2006) to assess the ecological niche of each lineage and to predict their potential range based on their georeferenced localities and environmental variables thereof. The occurrence data of *P. armandii* (excluding planted populations) were obtained from our field observations, literature (Liu et al., 2014, 2019; Ma, 1989), and from herbarium records from two sources: the Global Biodiversity Information Facility (GBIF, https://www.gbif.org/) and the National Specimen Information Infrastructure (NSII, www.nsii.org.cn). In total, 284 georeferenced points were obtained (Figure S7a).

Nineteen bioclimatic variables were acquired at 2.5 arc‐minute resolutions from WorldClim (www.worldclim.org) (Hijmans, Cameron, Parra, Jones, & Jarvis, 2005) for three periods: the present, the last glacial maximum (LGM, 18–21 ka), and the last interglacial period (LIG, 120–140 ka). To avoid model overfitting linked to correlated climatic parameters, only those seven variables that had low correlation coefficients with one another (*r* < 0.8) were retained for subsequent analysis (Table S5).

### 2.7 Niche comparison analyses on *G*‐spaces

To capture ecological differences in the niche occupied by each genetic lineage, likely reflecting local adaptation, niches of different lineages of *P. armandii* were compared in both geographic (*G*) and the environmental (*E*) spaces, because these two types of niche space have been shown to complement each other in niche comparison studies (Petitpierre et al., 2012). For each lineage, historical niche shifts in geographic distribution between the LIG, LGM, and present day were inferred, based on ENMs in geographic (*G*) space and using MAXENT with default settings. We limited our model extent to the distributional range of each regional lineage of *P. armandii* with a 200 km buffered zone, to eliminate the impact of background geographic area of the models on modeling results (Merow, Smith, & Silander, 2013).

Furthermore, to measure niche differences between lineages, we used ENMTOOLS v1.3 (Warren, Glor, & Turelli, 2008) to calculate the niche overlap statistic Schoener’s *D* (Schoener, 1968) and standardized Hellinger distance (calculated as *I*; Warren et al., 2008), where a value of 0 denotes no overlap and 1 indicates complete overlap. To test the null hypothesis that two lineages have identical ENMs, we used the niche equivalency test initially proposed by Warren et al. (2008) in ENMTOOLS. This test compares the observed scores of niche overlap statistics *D* and *I* with their null distribution generated with 100 pseudoreplicates (see Warren et al. (2008) for details).

### 2.8 Niche comparison analyses on *E*‐spaces

To assess the degree of niche overlap, we first assessed ENMs in spatial environmental (*E*) space using R packages (ECOSPAT, Di Cola et al., 2017). Following the approach initially proposed by Broennimann et al. (2012), principal components analysis (PCA) was used to translate occurrence and climate data into environmental axes (PCA‐env). Densities of points in multidimensional *E* space were then used to quantify ENM overlap, using the *D* and *I* statistics. The niche equivalency test was employed to test whether the environmental niche space of two lineages is identical using 100 pseudoreplicates.

Niche overlap between lineages can be characterized by niche unfilling, niche stability, and niche expansion (Broennimann et al., 2012; Guisan, Petitpierre, Broennimann, Daehler, & Kueffer, 2014; Petitpierre et al., 2012). Lineage A occupies a range termed A, and the assumption is made that lineage B has diverged from it and now occupies a range B. The term “unfilling” describes conditions within range A that do not overlap range B, whereas the term “niche stability” covers any areas of shared range between A and B, and “range expansion” describes those parts of range B that do not overlap with A. This classification provides additional information about the drivers of the niche dynamic between ranges (Di Cola et al., 2017; Petitpierre et al., 2012).

## 3 Results

### 3.1 CpDNA variation

The cpDNA *ycf1* fragment was successfully sequenced for all 466 sampled individuals of *P. armandii*, and a total of six chlorotypes were identified. Of these, three were common, with H1, H2, and H4 dominating populations from East Himalaya (EH), South Hengduan Mountains (SH), and Qinling‐Daba Mountains (QD), respectively; hence, these three regions were clearly defined as distinct by chlorotype data (Figure 1). H1 and H2 also occasionally occurred outside their dominant regions, whereas H4 was unique to QD (Figure 1).

All subsequent statements in this section refer to wild (not planted) material only, unless stated otherwise.

### 3.2 MtDNA variation

The concatenated mt DNA sequences of *nad*5 intron 1 and *nad*7 intron 1 comprised 1412 bp, and from this, four mitotypes were distinguished. The geographic distribution of these was highly structured, with M1, M2, M3, and M4 unique to QD, EH, east SH, and west SH, respectively (Figure S1).

### 3.3 Population genetic differentiation and structure

The AMOVAs revealed that 88.90% of overall cpDNA variation was between regions (QD, EH, and SH; Table 1). The coefficient of genetic differentiation of wild *P. armandii* populations was remarkably high (*G*_ST_ = 0.883 and *N*_ST_ = 0.930) (Table S6). According to mtDNA data, 55.21% of overall variation was between regions (Table 1). Conversely, nuclear (*AGP*6 and *LFY*) and microsatellite variation mainly occurred within populations (48.33%, 63.26%, and 79.48%, *F*_ST_ = 0.52, 0.37, and 0.21, respectively; Table 1) with all *F*_ST_ values highly significant (*p* < .001). According to microsatellite data, *F*_ST_ values between all three pairs of regions were highly significant (*p* < .001), with although EH was less differentiated from SH (0.17) than either was from QD (0.21 and 0.20, respectively).

**Table 1:** Hierarchical analyses of molecular variance (AMOVA) of *P. armandii* wild populations based on nuclear microsatellite, cpDNA, nDNA, and mtDNA genetic data

Source of variation	*df*	SS	VC	Variation ( %)	Fixation index
	Microsatellite markers				
EH					
Among populations	5	66.11	0.33	9.94	*F*_ST_ = 0.10***
Within populations	180	543.12	3.02	90.06	
SH					
Among populations	10	111.27	0.27	7.27	*F*_ST_ = 0.07***
Within populations	311	1060.85	0.97	92.73	
QD					
Among populations	22	180.74	0.19	4.71	*F*_ST_ = 0.05***
Within populations	503	1950.00	3.88	95.29	
Total populations					
Among groups	2	454.61	0.68	15.18	*F*_CT_ = 0.15***
Among populations within groups	37	358.13	0.24	5.34	*F*_SC_ = 0.06***
Within populations	994	3553.97	3.58	79.48	*F*_ST_ = 0.21***
Total	1033	4366.70	4.50		
	cpDNA				
EH					
Among populations	5	1.43	0.01	2.07	*F*_ST_ = 0.02
Within populations	57	13.33	0.23	97.93	
SH					
Among populations	10	0.72	0.001	1.46	*F*_ST_ = 0.02
Within populations	96	6.05	0.063	98.54	
QD					
Among populations	23	27.62	0.14	73.50	*F*_ST_ = 0.74***
Within populations	179	8.81	0.05	26.50	
Total populations					
Among groups	2	223.11	1.00	88.90	*F*_CT_ = 0.89***
Among populations within groups	38	28.44	0.08	6.89	*F*_SC_ = 0.62***
Within populations	322	15.75	0.05	4.21	*F*_ST_ = 0.96***
Total	372	267.30	1.13		
	*AGP*6				
EH					
Among populations	3	0.80	0.01	2.62	*F*_ST_ = 0.03
Within populations	30	6.55	0.22	97.38	
SH					
Among populations	10	18.27	0.15	21.56	*F*_ST_ = 0.22***
Within populations	108	57.88	0.54	78.44	
QD					
Among populations	21	163.33	0.81	30.94	*F*_ST_ = 0.31***
Within populations	142	256.32	1.81	69.06	
Total populations					
Among groups	2	172.52	0.79	31.23	*F*_CT_ = 0.31***
Among populations within groups	35	206.48	0.52	20.44	*F*_SC_ = 0.30***
Within populations	317	388.29	1.23	48.33	*F*_ST_ = 0.52***
Total	353	767.29	2.53		
	*LFY*				
EH					
Among populations	–	–	–	–	–
Within populations	–	–	–	–	
SH					
Among populations	1	0.41	‐0.01	‐1.92	*F*_ST_ =−0.02
Within populations	9	4.13	0.46	101.92	
QD					
Among populations	3	4.65	0.08	6.58	*F*_ST_ = 0.07
Within populations	18	20.17	1.12	93.42	
Total populations					
Among groups	4	12.27	0.46	32.02	*F*_CT_ = 0.32
Among populations within groups	2	5.06	0.07	4.72	*F*_SC_ = 0.07
Within populations	31	27.90	0.90	63.26	*F*_ST_ = 0.37***
Total	37	45.24	1.42		
	mtDNA				
Total populations					
Among groups	2	3,986.52	36.29	55.21	*F*_CT_ = 0.55***
Among populations within groups	19	4,948.60	29.44	44.79	*F*_SC_ = 1.00***
Within populations	187	0.00	0.00	0.00	*F*_ST_ = 1.00***
Total	205	8,935.13	65.72		

*df*, degrees of freedom; SS, sum of squares; VC, variance components; significance levels: *p < .05; **p < .01; and ***p < .001; 1000 permutations; *F*_CT_, divergence among groups within species; *F*_ST_, divergence within populations; *F*_SC_, divergence among populations within groups.

For the Bayesian analysis of population structure for wild material (*AGP*6 and *LFY* genes), Δ*K* indicated that the optimal value for *K* was 2 (Figure S2). At *K* = 2, one cluster comprised all EH and SH material plus some from QD, while the other comprised only material from QD (Figure S2). However, hierarchical analyses within these clusters detected no further genetic subdivision (Figure S3). Meanwhile, comparing with the full microsatellite dataset, a similar genetic pattern was obtained with rarefaction microsatellite dataset (Figure S4).

According to the STRUCTURE analysis of microsatellite data, examining both wild and planted populations together, the most likely number of genetic clusters was estimated at 2 (Figure 2). Populations from EH (1‐6) and SH (7‐20) formed one group, whereas the other group comprised all populations from QD (21‐52). When *K* = 3, the former cluster was further subdivided, separating EH (1‐6) from SH (7‐20) (Figure 2). Subsequent hierarchical analyses within each region did not show any further genetic subdivision (Figure S5). Additionally, when planted populations are excluded, the populations cluster in exactly the same way for both *K* = 2 and *K* = 3 (Figure S6b, c and e). Likewise, when assessing planted populations only, there is clear separation between the SH and QD populations (there are no planted populations in EH) (Figure S6a, d). All analyses revealed considerable rates of genetic admixture within each region, including between wild and planted populations, but not between regions (Figure 2 and Figures S3, S5, S6).

### 3.4 Genetic diversity within *P. armandii*

Overall cpDNA diversity of the wild *P. armandii* populations was high (*H*_T_ = 0.614), whereas the same measure within each region was lower (*H*s = 0.037, 0.133, and 0.050 for EH, SH, and QD, respectively) (Table S6). For planted populations, the *H*_T_ and *H*s values were 0.610 and 0.119, respectively (Table S6). Meanwhile, the total haplotype diversity (*H*_d_) and nucleotide diversity (*π*) values for *AGP*6 and *LFY* among wild populations were higher than those for planted populations (Table S7).

All microsatellite markers were highly polymorphic within *P. armandii* (Table S1). The expected (*H*_E_) and observed heterozygosity (*H*_O_) of the wild populations were 0.573 and 0.444, respectively. Interestingly, wild populations exhibited higher genetic diversity than the planted populations for each of four measures: effective allele number, expected heterozygosity, observed heterozygosity, and Shannon’s Information Index. The Kruskal–Wallis tests did not detect a significant difference in genetic variation between planted and wild populations (Table S8), but based on all evidence available, overall variation was probably higher in the wild populations. On average, QD was more polymorphic than the other two regions.

### 3.5 Genetic migration among groups

The BAYESASS analysis detected recent genetic migration from EH to SH (*m* = 0.024) and from SH to QD (*m* = 0.021) (Table S9). Additionally, Migrate‐n identified historical asymmetric gene flow (*N*m) between all three regions, with the greatest *N*m between QD and SH (10.886; Table 2). Estimated population sizes according to Bayesian modes (Table 2), with 95% confidence interval (CI), were 1.755 (95% CI: 1.677‐1.838) for EH, 1.064 (95% CI: 1.031‐1.097) for SH, and 2.564 (95% CI: 2.496‐2.634) for QD.

**Table 2:** Migrate‐n Bayesian modes of effective population size (*θ* = 4Neμ) and bidirectional gene flow (*N*m = immigrants per generation). Numbers in parentheses are the lower 5% and upper 95% of posterior distribution

Source	*θ*	*N*m (mode) into recipient populations		
		EH	SH	QD
EH	1.755 (1.677–1.838)	–	3.127 (2.904–3.360)	6.451 (6.187–6.725)
SH	1.064 (1.031–1.097)	2.669 (2.477–2.872)	–	7.878 (7.594–8.167)
QD	2.564 (2.496–2.634)	7.298 (6.990–7.616)	10.886 (10.461–11.319)	–

EH, East Himalaya; SH, South Hengduan Mountains; QD, Qinling–Daba Mountains.

### 3.6 Evolutionary dynamics and changes in effective population size

In the DIYABC analysis, the posterior probability for scenario 3 (with 95% CI) was 0.964 (95% CI: 0.955‐0.972), much higher than for scenarios 1 (0.001; 95% CI: 0.000‐0.002), 2 (0.0101; 95% CI: 0.0058‐0.0144), or 4 (0.025; 95% CI: 0.019‐0.032). The median values of the effective population sizes of EH, SH, and QD were 3.60 × 10^5^, 1.08 × 10^6^, and 1.32 × 10^6^, respectively, whereas *N*_A_ was 9.78 × 10^4^ (Table 3). The estimated median time of divergence between EH and SH (t1) was 2.22 × 10^5^ generations ago, whereas QD diverged from the common ancestor of these 6.42 × 10^5^ generations ago (t2).

**Table 3:** Demographic approximate Bayesian computation (ABC) models for *P. armandii*

Parameters	*N*1	*N*2	*N*3	*N*4	*N*A	t1 (generations)	t2 (generations)	*µ*	*P*
Regions referred to	EH	SH	QD	EH+SH	EH+SH+QD	EH from SH	EH+SH from QD		
Median	3.60E+05	1.08E+06	1.32E+06	3.40E+05	9.78E+04	2.22E+05	6.42E+05	1.21E−06	0.5
Lower_bound	1.48E+05	5.11E+05	7.15E+05	6.26E+04	1.00E+04	8.58E+04	2.93E+05	4.89E−07	0.254
Upper_bound	7.19E+05	1.76E+06	1.86E+06	8.48E+05	1.89E+05	3.73E+05	9.51E+05	3.50E−06	0.685

*N*1, effective population size of EH; *N*2, effective population size of SH; *N*3, effective population size of QD; *N*4, effective population size of ancestral population of EH and SH; *N*A, effective population size of ancestral population; t1, time since divergence between EH and SH; t2, time since divergence between QD and ancestral population of EH and SH; µ, mutation rate (per generation per locus).

The microsatellite data for the EH and SH regions fitted best the contraction–expansion model, with posterior probability 0.308 (95% CI: 0.261–0.355) and 0.447 (95% CI: 0.431–0.463), respectively. EH material contracted 1.93 × 10^5^ generations or 4.83 Ma ago and then expanded 6.51 × 10^4^ generations or 1.63 Ma ago. Both happened slightly earlier in SH, with contraction 2.14 × 10^5^ generations or 5.35 Ma ago, then expansion 7.93 × 10^4^ generations or 1.98 Ma ago. QD material best fitted a continuous expansion model, with probability 0.448 (95% CI: 0.398‐0.498) (Figure 3; Table S10).

### 3.7 Lineage divergence time based on chloroplast genome

A molecular phylogenetic tree was constructed using completed chloroplast genome sequences from the three lineages within *P. armandii* and outgroup species (Figure 4). The divergence time of *P. armandii* from *P. monticola* was estimated at 15.2 Ma (95% HPD: 6.6–22.2). Within *P. armandii*, the three regional lineages EH, SH, and QD each formed strongly supported clades, and the first divergence was that of QD around the Tortonian period 9.0 Ma (95% HPD: 4.0–14.5), with divergence between EH and SH occurring later, 6.7 Ma (95% HPD: 2.8–12.0).

### 3.8 Ecological niche modeling

The predicted model for the present potential range of *P. armandii* generated with MAXENT was fairly congruent with the current distribution of the species (Figure S7), but this was more accurately predicted when considering the three lineages separately, with AUC (area under the curve) values ≥0.9 (Figure S8). Comparing with its current range, the areas of suitable habitat in the EH region, QD, and adjacent areas were much wider during the LGM. Conversely, the species’ range was more restricted during the LIG than at present (Figure S7). Similar patterns were obtained when each regional lineage was examined separately, with each having the largest range during the LGM and the smallest during the LIG (Figure S8).

**Figure 5:**
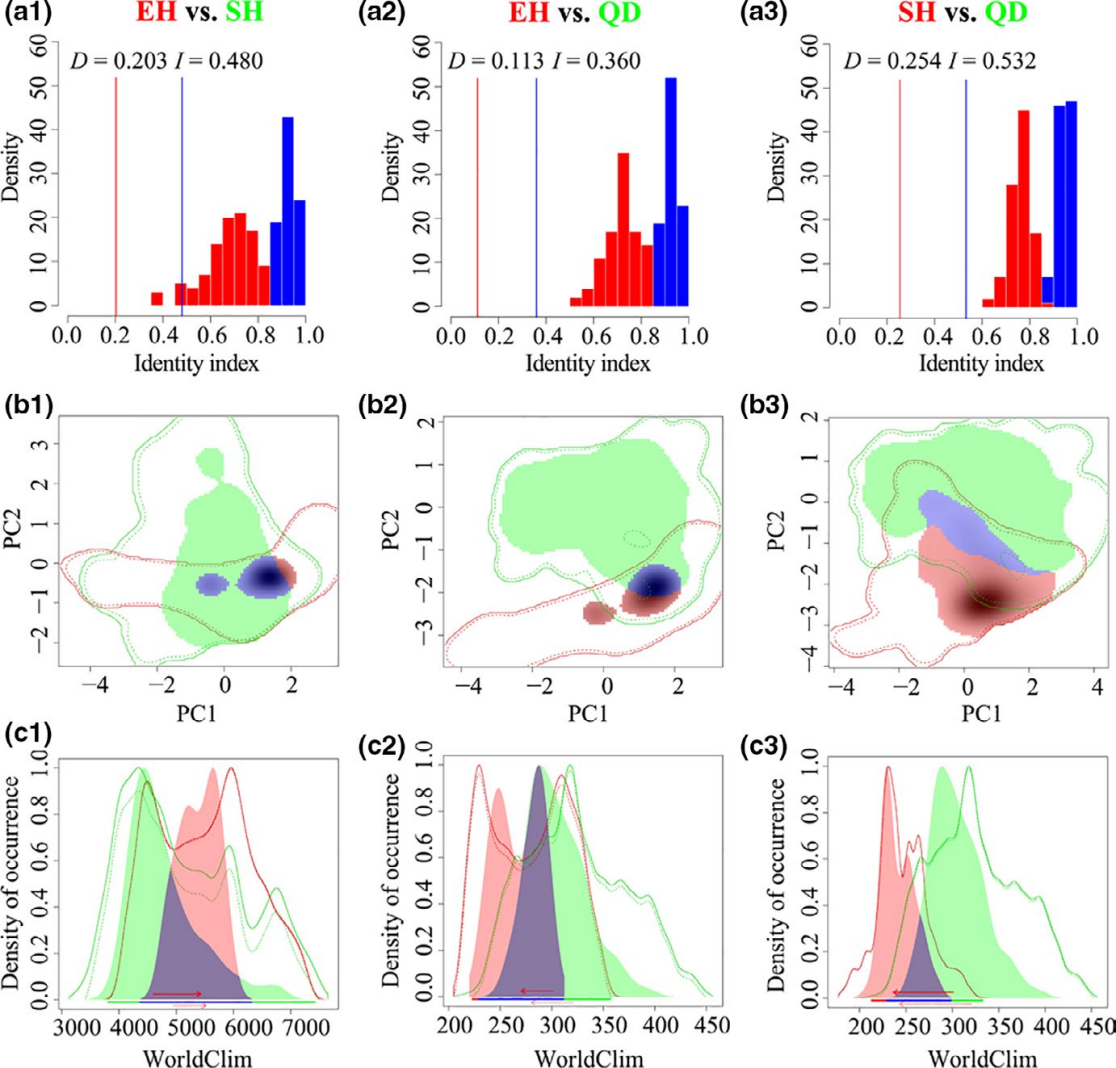
(a1–a3) Niche overlaps of *P. armandii* based on pairwise comparisons among the three lineages across climatic space. For each analysis, the lineages in red and green are lineages A and B in the analysis, respectively, with overlapping densities between ranges shown in violet. The solid and dashed contour lines delimit the 100th and 75th quantiles, respectively, of the density at the available climate. (b1–b3) Densities of available climates and *P. armandii* occurrences based on pairwise comparisons; the horizontal bars show the components of niche dynamics present along the x‐axis: unfilling (green), stability (violet), and expansion (red). The solid arrows represent the shift direction of the niche centroid between the designated lineages A and B, and the dashed arrows represent the shift direction of the average available environmental conditions between ranges. (c1–c3) Niche equivalency test for each comparison based on Schoener’s *D* statistic (Schoener, 1968), Warren’s *I* statistic (Warren et al., 2008), and Maxent predictions. Bars indicate the null distributions of *D* and *I*

Niche overlap statistics demonstrated that each lineage occupied a distinct niche (Figure 5a) based on *G* space. ENMTOOLS showed that empirically observed values for *I* and *D* were significantly lower than those expected from pseudoreplicated datasets in all paired analyses (EH vs. SH, EH vs. QD, and SH vs. QD, *p* < .01) (Figure 5a). However, no such regional difference was significant (*p* > .05, Figure S9a, b) in the PCA‐env analysis, which hence detected no difference in niches between regions.

In pairwise comparisons of regions, first two axes of the PCA‐env analyses accounted for 88.95%, 86.92%, and 84.05% of the variation in EH vs. SH, EH vs. QD, and SH vs. QD comparisons, respectively (Figure S9c1‐c3). Climatic niche overlap between the SH and QD populations was the highest (*D* = 0.149), whereas niche overlap between EH and QD was the lowest (*D* = 0.086) (Figure 5b1–b3). Concerning climatic niche partitioning across the three comparisons between regional lineages’ climatic niches, “unfilling” niches account for 44.0%‐80.8%, whereas “stable” niches (shared ranges between lineages) account for 24.2–88.6%, and “range expansion” niches account for 11.4–75.8% (Figure 5c1–c3; Table S11). The proportion of “unfilling” niches was much higher for between EH and SH (80.8%), and between SH and QD (70.7%), than between EH and QD (40%) (Table S11).

## 4 Discussion

In this study, we employed an integrative approach to address the evolutionary legacy of widespread afforestation of *Pinus armandii* within its native range, in China, using large‐scale population genetics and phylogenomic analysis. We identified three intraspecific lineages, each occupying distinct ecological niches (Figure 5). These diverged around the late Miocene (Figure 4) during a period of massive uplifts of the Hengduan Mountains and intensification of Asian Summer Monsoon (Clift & Webb, 2019; Wang et al., 2012). The oldest successful plantings of *P. armandii* date from the 1980s, and all plantations examined were probably sourced from the same region, as had been indicated for those planted populations previously examined (Ma, 1989). Therefore, planted material presents little or no probability of allowing between‐region gene flow.

### 4.1 Lineages within *P. armandii* and their biogeographic history

Despite widespread human influences, native forests might retain genetic signals from their past distribution, due to natural regeneration of local stock in combination with the long life of individual trees (Petit & Hampe, 2006), thereby allowing evolutionary backgrounds of natural ranges to be detected. Our data separated three geographic lineages within *P. armandii* (Figures 1, 2, 4; Figure S7). Some admixture, and hence limited gene flow between regions EH and SH, was indicated by the presence in populations p6 (EH), p13 and p14 (SH) of the common haplotype from the other region. At the boundary between these regions, ecological conditions might form a gradient facilitating gene flow, whereas anthropogenic disturbances like deforestation might have promoted it, for example, where regeneration occurs but some distance away from surviving native populations. Furthermore, population p30, which is a southwestern geographic outlier within QD, contains mainly haplotype H1 from EH. There were also minor discrepancies between groupings based on microsatellite loci and those indicated by STRUCTURE analysis of nuclear genes (*AGP*6 and *LFY*) (Figure 2; Figure S2), which may be caused by the two types of genetic markers having different demographic histories and mutation rates (Petit, Duminil, Fineschi, Hampe, & Vendramin, 2005).

These three ecogeographic lineages had been suggested by previous molecular data (Liu et al., 2014), but the greatly increased sampling and genomic coverage of the current study clearly resolved their full ranges for the first time, and also their relationships. The most northerly lineage, QD, diverged first, around the middle Miocene (ca. 9.0 Ma; Figure 4). Later, during the late Miocene (ca. 6.7 Ma; Figure 4), EH diverged from SH, and both lineages are likely to have been greatly affected by the orogeny of the Hengduan Mountains on the eastern margin of Tibetan Plateau from the late Miocene onwards (Clark et al., 2005; Wang et al., 2012; Yang et al., 2017). Furthermore, from then onwards all three lineages would have been profoundly affected by barriers to gene flow such as the parallel massive mountains and deeply carved valleys in Hengduan Mountains; these barriers have promoted allopatric divergence within other conifers (e.g., *Taxus wallichiana* (Liu et al., 2013); *Cupressus* (Xu et al., 2010)).

Where mountain building and local climate change happen in concert, this will accelerate niche divergence (Antonelli et al., 2018). Niche divergence was proposed as the causes for divergence within *Taxus wallichiana* (Liu et al., 2013) and *Roscoea* (Zhao, Gugger, Xia, & Li, 2016) in the Hengduan Mountains. Hence, mountain uplifts there coupled with the intensification of the Asian Summer Monsoon during the Miocene (An et al., 2014; Clift & Webb, 2019) likely caused niche differences between EH, SH, and QD, driving diversification between material from different regions. According to fine‐resolution climatic data analysis, these intraspecific lineages occupy distinct ecological niches from one another (Figure 5), confirming earlier findings of differing ecology and cold tolerance between regions (Ma, 1989). Considering past ranges, ecological niche modeling of the distribution ranges of *P. armandii* confirmed the trend of an increase in area of occupancy between the LIG and LGM (Figure S7), probably due to the increased availability of cold habitats. This might have reinforced the divergence of the three lineages through differential adaptation to their respective environments, as detected for other Alpine conifers, that is, *Picea likiangensis* (Li et al., 2013) and *Taxus wallichiana* (Liu et al., 2013). Meanwhile, the demographic history of *P. armandii* is complex according to ABC simulations, involving population expansions followed by strong bottlenecks and expansions from the late Miocene to Pleistocene. In particular, the expansion of lineages EH, SH, and QD began at 1.63 Ma, 1.98 Ma and 2.42 Ma, respectively, all in the early Pleistocene and well before the LIG (0.14–0.12 Ma). The fact that a moderately cold climate has prevailed on the QTP before the LIG will have provided opportunities for each of the three lineages to have continued its range expansion throughout the LIG, as previously proved in *Picea likiangensis* (Li et al., 2013).

### 4.2 Origin of planted material

Although previous studies have carried out provenance trials in some plantations of *P. armandii* (Ma, 1989, 1992), our study is the first to examine the full native and planted range of the species. Throughout its range, we found that at least nine of the 11 planted populations matched nearby wild populations in terms of both the dominating haplotype and genetic similarity for other markers (Figures 2, 3) and hence were sourced within the same region, as tended to be found for individual plantations by previous work (Ma, 1989, 1992). Of the other two populations, p23 in QD has the chlorotype H1, which is mainly from the SH region; however, it resembles other QD populations for nuclear data, and moreover, the chlorotype H1 is also fixed in wild population p48, which is by far the closest geographically to p23 (Figures 1, 2; Table S1). Hence, much the likeliest origin for p23 is that it was sourced very locally, from the nearest wild material, implying that planted material, even within a region, does not all originate from a common pool of cultivated stock.

Planted population p8 from EH has a 1:1 ratio of haplotypes H1 and H2, which are, respectively, rare and dominant in EH, whereas H1 is far commoner in SH (Figure 1). As with p23, however, this population resembles local wild populations for nuclear data (Figure 2). Hence, it was probably sourced from within EH, but not from the nearest sampled wild populations, p9 and p10, which do not contain haplotype H1. Instead, it might have been sourced from a more distant EH population such as p13 or p14 (both > 500 km away), or an unsampled or extinct population closer by. The high proportion of H1 here might be a chance outcome of a bottleneck event (see below). Crucially, while p23 indicates that some planted populations were probably sourced very locally even within a region, p8 indicates that this might not apply for all of them.

Because planted populations examined were all sourced from within the region where they occurred, these appear to have little potential for allowing gene flow between regions. This also means that, contrary to general expectations, the inclusion of planted populations did not obscure the genetic evidence of phylogeographic structure within *P. armandii*. The potential remains for planted material to promote within‐region gene flow, especially where plantations are some distance from their source, and hence perhaps also local genetic swamping. However, for genetic swamping to occur, local adaptation of genotypes, and fitness variation between populations, must be small (Anttila et al., 2000; Hufford & Mazer, 2003). To test this possibility, within‐region variation in climatic niche needs investigation alongside whether planted material is sometimes nonlocally sourced within a region.

To detect gene flow between planted and wild material, seedlings and saplings need to be sampled as there has not been time for two generations to be completed since planting (at least from plantations sampled). Moreover, markers must be developed that detect within‐region geographic structure, through which introgression from elsewhere within the region, via material planted away from its source, can be detected.

Lower levels of diversity in planted than wild accessions (Tables S1, S6, S7, and S8) potentially indicate bottleneck events during or before establishment. However, planted population p31 was the only population from which four haplotypes were detected, so at least some planted populations seem to remain genetically diverse, and there may be variation in how planted seed is sourced, as well as from where.

### 4.3 Anthropogenic and climatic impacts on the source of planted material

The process of introduction of tree species around the world is generally informed by niche concept and the principle of climatic niche similarity (Li, Zhang, Huang, Wen, & Du, 2018), giving rise to a site‐species matching principle. Within *P. armandii,* earlier transplant experiments revealed strong local adaptation apparently driven by regional differences in mean annual temperature and extreme minimum temperature (Tang, 1995), leading to material from different regions differing significantly in cold resistance (Ma, 1989). The current study backed this up with niche equivalency test comparisons, indicating that the lineages occupy nonidentical ecological niches (Figure 5b) and distinct climatic regimes, with little overlap (Figure S8).

However, there was still some overlap in the niche space among lineages, in particular between SH and both EH (*D* = 0.103, *I* = 0.286) and QD (*D* = 0.149, *I* = 0.101) (Table S11) based on PCA‐env niche predictions, which is not surprising considering their geographic proximity (Figure S7a). Despite this limited overlap, habitat‐specific adaptation in different lineages might act as an ecological barrier preventing immigrants from surviving and reproducing in alternate habitats, which further strengthens their genetic differentiation (Liu et al., 2019). The consequent risk of maladaptation might have influenced the decision to plant locally sourced material, according to the site‐species matching principle, during afforestation programs for *P. armandii* in China. However, while provenance studies have likely led to deliberate site‐species matching in some instances (Ma, 1989, 1992), local planting in some places might have come about through trial and error, or because planted seed was sourced from nearby trees merely for convenience. Attempts at afforestation using *P. armandii* in central China during the 1960s and 1970s were unsuccessful, due to material of one lineage (SH) being planted within the range of another (QB), leading to the dieback of trees in winter frost (Ma, 1992; Wang & Hong, 2004). To avoid such losses, material for planting should always be sourced within the range of the local *P. armandii* lineage, and not outside it, to ensure good performance. While this might be enough to ensure plantation survival, local microadaptation within lineages may also occur (Mahony et al., 2019), and so performance is likely to be further enhanced by provenance trials and transplant experiments, although these are time‐consuming and expensive.

### 4.4 Implications for afforestation policy

To date, there has been little emphasis on assessing the genetic legacy of a widespread afforestation program, despite growing investment in large global afforestation projects (Payn et al., 2015). Our results build upon existing phylogeographic and historical evidence of coniferous tree species, to substantially advance our understanding of the contemporary genetic impact of past widespread afforestation. We also show that ecological niche models can be used to predict areas with suitable habitat and climate for introduction and plantation around the world. Despite concern that translocation of individuals between regions with different lineages might facilitate within‐species introgression, we found that instead the consequence of this, at least in *P. armandii*, is more likely to be failure of plantations due to maladaptation. Earlier work provides examples of this: Southern material could barely survive northern winters, whereas northern material grew very slowly in the south (Ma, 1989, 1992). Although the site‐species matching principle has clearly worked for *P. armandii* so far, shifts in potential distribution ranges due to future climate change in *P. armandii* (Zheng, Gao, & Zhang, 2017) might alter this in the future, and material originally from warmer or wetter regions, such as the SH lineage, might become more suitable for future afforestation programs in the QD and EH lineages range.

Intraspecific genetic variation is essential for species adaptation and survival and can have profound effects on ecological processes, across communities and even ecosystems (Hughes, Inouye, Johnson, Underwood, & Vellend, 2008; Jordan, Breed, Prober, Miller, & Hoffmann, 2019). Neglecting this factor while implementing afforestation policy may create big risks for forest health in the future. Low plantation diversity can result from bottlenecks, or from planted seed originating from small isolated remnant populations that lack adequate genetic diversity (Durka et al., 2017). Most planted forests are either monocultures or compositionally simple mixed forests, and few comprise much intraspecific genetic diversity, making them susceptible to abiotic and biotic threats exacerbated by global change (Verheyen et al., 2016). Overall, to create forests that form sustainable ecosystems, it is important to know the origin of planted material, and there is an urgent need to comprehensively evaluate the genetic effect of afforestation by other dominant afforestation tree species, based on both genetic and ecological perspectives.

## AUTHOR CONTRIBUTIONS

Z‐HL and JL conceived the study. YJ and JZ performed the experiments. Z‐HL, YJ, JZ, L‐MG, G‐FZ, and G‐FZ contributed materials and analysis tools. Z‐HL, JL, R‐IM, and YJ wrote the manuscript. Z‐HL, JL, R‐IM, and YJ revised the manuscript. All authors approved the final version of the manuscript.

## Supporting information

Supplementary Material

## Data Availability

Microsatellite datasets are available on the DRYAD: https://doi.org/10.5061/dryad.3ffbg79dx. DNA sequences are deposited in GenBank under accession numbers MN305672‐MN305686, MN382181‐MN382262, and MT515758‐MT515763.

## Appendix 1 Materials and Methods

### Assembly and annotation of chloroplast genome

The raw sequencing reads were trimmed using the NGSQC TOOLKIT v.2.3.3 with the default parameters (Patel & Jain, 2012). After trimming of adapters, low‐quality sequences (quality value ≤ 20), and reads with more than 30% low‐quality bases, the resulting clean reads were used to perform referenced‐guided assembly using MIRA v4.0.2 (Chevreux et al., 2004) and MITOBIM v1.7 (Hahn, Bachmann, & Chevreux, 2013) with *P. armandii* (NC_029847) as reference. The assembled plastome was annotated using GENEIOUS v9.1.4 (Biomatters Ltd, Anzac Avenue, New Zealand) by comparing the plastome of *P. armandii* (NC_029847).

### DNA sequence analysis

Chloroplast and mitochondrial haplotypes were identified using DNASP v5.00.01 (Librado & Rozas, 2009), and a haplotype network was constructed using NETWORK v4.2.0.1 (Bandelt, Forster, & Röhl, 1999).

Population genetics parameters of cpDNA sequences were estimated using DNASP v5.00.01 (Librado & Rozas, 2009) and PERMUT v1.2.1 (Pons & Petit, 1996). In addition, we used DNASP v5.00.01 (Librado & Rozas, 2009) to estimate the total haplotype diversity (*H*_d_), Watterson’s theta (*θ*_W_), and nucleotide diversity (*π*) (Nei, 1987) for the two nuclear genes.

We then performed a hierarchical analysis of genetic differentiation for cpDNA, mtDNA, and nuclear markers using an analysis of molecular variance (AMOVA), which partitioned total molecular variance within and among populations with 1,000 permutations and was implemented in ARLEQUIN v3.5 (Excoffier & Lischer, 2010). This was performed once for the whole species and then again separately for each of the three regions.
