# Supplementary material for: Evolutionary legacy of a forest plantation tree species (Pinus armandii): Implications for widespread afforestation
Source: Evol Appl. 2020 Jul 27;13(10):2646–62. doi: 10.1111/eva.13064 (PMC7691453; doi:10.1111/eva.13064)
Supplement: Supplementary file 1 — Supplementary Material [file EVA-13-2646-s001.docx]

**Supporting information**

**Supporting Tables (eleven Tables, Table S1-11)**

**Supporting figures (eight figures, Fig. S1-9)**

**Supporting Tables**

Table S1 Details of sample locations, sample sizes and descriptive statistics of genetic variability based on 12 microsatellite loci for the 52 sampled populations of *P. armandii* examined.

Table S2 PCR primers used in this study.

Table S3 Information concerning the 24 complete chloroplast genomes of *Pinus* used for phylogenomics analysis in the present study.

Table S4 Prior distributions for model parameters used in divergence model comparisons (Fig. 3a).

Table S5 The seven bioclimatic variables that were used in ecological niche modelling.

Table S6 Genetic diversity of cpDNA in the three distinguished lineages of *P. armandii*.

Table S7 Nucleotide variation and haplotype diversity of *P. armandii* across the nuclear loci *AGP*6 and *LFY.*

Table S8 Kruskal–Wallis test for genetic diversity differences of microsatellite loci between wild and planted populations of *P. armandii*.

Table S9 Estimation of rates of contemporary gene flow per generation among three lineages (EH, SH, and QD) using the programs BAYESASS v3.0.

Table S10 Posterior median estimate and 95% highest posterior density interval for demographic parameters of a contraction-expansion model based on the nuclear microsatellite data of the three groups.

Table S11 Results of pairwise niche overlap estimates and the proportion of niche change among three lineages.

**Supporting figures**

Fig. S1 Geographic distribution and network tree based on haplotypes detected in *P. armandii* using combined data from two mitochondrial fragments (*nad*5 intron 1 and *nad*7 intron 1).

Fig. S2 Bayesian inference analysis of nuclear data for determining the most likely number of clusters (*K*) for *P. armandii*. The distribution of the likelihood L(*K*) values (a) and Delta*K* values (b) are presented for *K* = 1–15 (10 replicates). STRUCTURE plots (c) are presented for the best-performing value, *K* = 2.

Fig. S3 Bayesian inference analysis of nuclear data for each group separately. STRUCTURE plots are presented for *K* = 2 and *K* = 3. Red numbers denote planted populations.

Fig. S4 Bayesian inference analysis of a rarefaction microsatellite dataset which include same number of individuals as nuclear gene dataset for determining the most likely number of clusters (*K*) for *P. armandii*. The distribution of the likelihood L(*K*) values (a) and Delta*K* values (b) are presented for *K* = 1–15 (10 replicates). STRUCTURE plots (c) are presented for the *K* = 2 and *K* = 3.

Fig. S5 Bayesian inference analysis of microsatellite data for each group separately. STRUCTURE plots are presented for *K* = 2–6 (a). The distribution of Delta*K* values are presented for *K* = 1–10 (10 replicates) for EH (b), SH (c) and QD (d). Red numbers denote planted populations.

Fig. S6 (a) Genetic structure analyses in planted and wild populations of *P. armandii.* STRUCTURE plots in planted populations of *P. armandii* are presented for the best-performing value, *K* = 2; (b-c) STRUCTURE plots in wild populations of *P. armandii* are presented for *K* = 2 and *K* = 3; (d) The distribution of Delta*K* values are presented for *K* = 1–15 (10 replicates) in planted populations of *P. armandii*; (e) The distribution of Delta*K* values are presented for *K* = 1–15 (10 replicates) in wild populations of *P. armandii*.

Fig. S7 (a) Map showing a total of 284 distribution records for *P. armandii* sourced from the Global Biodiversity Information Facility (GBIF, <http://www.gbif.org/)>, the National Specimen Information Infrastructure (NSII, <http://www.nsii.org.cn/)>, field work, and literature (Ma, 1989; Liu et al., 2014; Liu et al. 2019). Blue, red and black symbols represent populations of EH, SH and QD respectively. (b-d) Suitable ranges for *P. armandii* during three time periods as predicted by MAXENT: (b) Present, based on conditions *c.* 1950–2000; (c) LGM, last glacial maximum (*c.* 21 000 a BP), and (d) LIG, last interglacial (c. 120 000–140 000 a BP). Red colours indicate higher probabilities of suitable climatic conditions.

Fig. S8 Predicted potential distributions of the three lineages (EH, SH and QD) in *P. armandii* based on ecological niche modelling.

Fig. S9 (a1-3) Niche equivalency test for each comparison based on Schoener’s *D* statistic (Schoener 1968) and PCA-env predictions; (b1-3) Niche equivalency test for each comparison based on Warren’s *I* statistic (Warren et al. 2008) and PCA-env predictions; (c1-3) Correlation circle showing the contribution of climatic variables on each axis.

Table S1 Details of sample locations, sample sizes and descriptive statistics of genetic variability based on 12 microsatellite loci for the 52 sampled populations of *P. armandii* examined.

| Pop. | Location | Longitude (E) | Latitude (N) | Altitude (m) | Plantation/Wild | Estimate population age | Planting time | Area (ha) | Reference | Annual average rainfall  (mm) | Annual average temperature  (℃) | Coldest month temperature  (℃) | Individuals for SSR/ cpDNA/mtDNA/nuclear gene/chloroplast genome sequences (*N*) | Microsatellite | | | | |
| --- | --- | --- | --- | --- | --- | --- | --- | --- | --- | --- | --- | --- | --- | --- | --- | --- | --- | --- |
|  |  |  |  |  |  |  |  |  |  |  |  |  |  | *N*_A_ | *N*_E_ | *I* | *H*_O_ | *H*_E_ |
| 1 | Linzhi, Xizang | 94.77 | 29.86 | 2435 | Wild | 60-70 |  |  |  | 650 | 9.1 | 1.7 | 18/11/10/6/ | 3.9167 | 2.5995 | 0.9436 | 0.3562 | 0.5102 |
| 2 | Chayu, Xizang | 97.29 | 29.11 | 3200 | Wild | 40-45 |  |  |  | 793.9 | 12.1 | 5.3 | 20/12/0/0/ | 4.0833 | 2.7178 | 0.9798 | 0.3693 | 0.5303 |
| 3 | Bomi, Xizang | 95.72 | 29.84 | 3055 | Wild | 75-80 |  |  |  | 977 | 9 | 0.8 | 19/11/10/0/ | 3.6667 | 2.0863 | 0.8305 | 0.3596 | 0.4587 |
| 4 | Lulang, Xizang | 94.77 | 29.86 | 2595 | Wild | 55-65 |  |  |  | 650 | 9.1 | 1.7 | 12/9/10/10/ | 3.9167 | 2.2513 | 0.89 | 0.3889 | 0.49 |
| 5 | Yupu, Xizang | 96.3 | 29.62 | 3218 | Wild | 88-90 |  |  |  | 977 | 9 | 0.8 | 14/11/10/13/1 | 3.3333 | 2.1301 | 0.7901 | 0.3631 | 0.4579 |
| 6 | Nixi, Yunnan | 99.49 | 28.07 | 3135 | Wild | 63-65 |  |  |  | 503 | 6.2 | 8.5 | 10/9/0/10/1 | 4.3333 | 3.2266 | 1.1456 | 0.4417 | 0.6272 |
|  | **EH** |  |  |  |  |  |  |  |  |  |  |  |  | **3.819** | **2.444** | **0.919** | **0.375** | **0.791** |
| 7 | Lijiang, Yunnan | 100.2 | 27 | 2752 | Wild | 40-48 |  |  |  | 935 | 12.9 | 6.6 | 24/10/3/6/ | 5.1667 | 2.751 | 1.079 | 0.3105 | 0.5478 |
| 8 | Huize, Yunnan | 103.35 | 26.09 | 2884 | Plantation （afforestation） | 34-35 | 1979 | 2700 | Feng, Tang & Cui 2007 | 817.7 | 13 | 5.9 | 20/10/0/10/ | 5 | 2.8911 | 1.1045 | 0.3542 | 0.5682 |
| 9 | Xuanwei, Yunnan | 104.19 | 26.28 | 2311 | Plantation （afforestation） | 34-45 |  |  | Field investigation | 1200 | 13.7 | 6.5 | 20/8/0/10/ | 4.4167 | 2.5877 | 0.9467 | 0.327 | 0.4907 |
| 10 | Changning, Yunnan | 99.87 | 25.04 | 2344 | Plantation （afforestation） | 26-30 |  |  | Field investigation | 625 | 15.1 | 8.6 | 20/10/0/10/ | 4 | 2.2434 | 0.8658 | 0.2833 | 0.4529 |
| 11 | Shuilu, Yunnan | 99.81 | 24.98 | 2136 | Wild | 65-70 |  |  |  | 1259 |  |  | 20/10/0/0/ | 4.1667 | 2.4402 | 0.8671 | 0.3083 | 0.4423 |
| 12 | Jiulong, Sichuan | 102.03 | 29.38 | 2873 | Wild | 33-36 |  |  |  | 1100 | 9.1 | 1.6 | 16/10/0/16/ | 5.5 | 3.2119 | 1.2048 | 0.3594 | 0.6022 |
| 13 | Ninglang, Yunnan | 100.85 | 27.29 | 2248 | Wild | 34-45 |  |  |  | 920 | 12.6 | 4.5 | 10/9/0/0/ | 4.9167 | 3.231 | 1.2222 | 0.5 | 0.6452 |
| 14 | Lanping, Yunnan | 99.02 | 26.95 | 2432 | Wild | 60-64 |  |  |  | 980-1010 | 11.3 | 3.9 | 10/10/0/12/1 | 3.75 | 2.5679 | 0.9383 | 0.4 | 0.5088 |
| 15 | Mianning, Sishuan | 102.22 | 28.53 | 1772 | Wild | 50-55 |  |  |  | 1095 | 14.1 | 7 | 10/10/10/11/ | 3.75 | 2.7721 | 1.0174 | 0.4167 | 0.5772 |
| 16 | Yanyuan, Sichuan | 101.51 | 27.4 | 2563 | Wild | 45-48 |  |  |  | 855.2 | 12.3 | 5.4 | 7/7/0/14/1 | 3.8333 | 3.0569 | 1.0147 | 0.3929 | 0.5668 |
| 17 | Shangri-la, Yunnan | 99.64 | 27.9 | 3282 | Wild | 60-66 |  |  |  | 268-945 | 6.3 | -1.6 | 8/8/7/0/ | 4.25 | 3.1663 | 1.1362 | 0.4836 | 0.6364 |
| 18 | Baihua ridge, Yunnan | 98.76 | 25.31 | 2531 | Wild | 56-60 |  |  |  | 700—2100 | 16.2 | 9.8 | 21/10/0/14/1 | 4.6667 | 2.6475 | 1.0731 | 0.4563 | 0.5682 |
| 19 | Lushui, Yunnan | 98.7 | 25.97 | 2826 | Wild | 65-70 |  |  |  | 1420 | 17 |  | 15/13/0/10/1 | 4.1667 | 2.5244 | 1.0236 | 0.4396 | 0.5567 |
| 20 | Dali, Yunnan | 99.29 | 25.87 | 2736 | Wild | 45-55 |  |  |  | 1600 | 16.2 | 8.9 | 20/10/0/14/1 | 5.5833 | 2.9562 | 1.2503 | 0.4819 | 0.6309 |
|  | **Planted population** |  |  |  |  |  |  |  |  |  |  |  |  | **4.444** | **2.569** | **0.969** | **0.321** | **0.491** |
|  | **Wild population** |  |  |  |  |  |  |  |  |  |  |  |  | **4.515** | **2.842** | **1.073** | **0.412** | **0.547** |
|  | **SH** |  |  |  |  |  |  |  |  |  |  |  |  | **4.500** | **2.738** | **1.051** | **0.393** | **0.535** |
| 21 | Neixiang, Henan | 111.94 | 33.52 | 1690 | Wild | 66-70 |  |  |  | 819.1 | 15.5 | 3.5 | 15/10/10/20/ | 4.9167 | 2.8812 | 1.178 | 0.3528 | 0.6136 |
| 22 | Zhashui, Shaanxi | 109.38 | 33.67 | 1370 | Wild | 80-90 |  |  |  | 742 | 12.2 | 1.5 | 17/10/5/16 | 5.3333 | 3.2641 | 1.2014 | 0.47 | 0.6082 |
| 23 | Shennongjia, Hubei | 110.47 | 31.72 | 1825 | Plantation （afforestation） | 65-75 |  |  | Field investigation | 800-2500 | 12.3 | 2.8 | 16/10/0/8/ | 5.9167 | 3.4302 | 1.3629 | 0.4444 | 0.6916 |
| 24 | Liangdang, Gansu | 106.3 | 33.91 | 962 | Wild | 45-55 |  |  |  | 600-700 | 10-11 | 0.9 | 20/10/3/10/ | 6.25 | 3.6033 | 1.3396 | 0.4053 | 0.6497 |
| 25 | Shanyang, Shaanxi | 110.03 | 33.4 | 953 | Wild | 55-60 |  |  |  | <700 | 13.1 | 2.2 | 18/10/0/10/ | 5.25 | 3.0354 | 1.2097 | 0.3821 | 0.6156 |
| 26 | Meixian, Shaanxi | 108.13 | 33.92 | 1615 | Wild | 55-60 |  |  |  | 609.5 | 12.8 | 0.7 | 20/10/10/10/ | 5.5833 | 3.4227 | 1.2382 | 0.4511 | 0.6161 |
| 27 | Xunhua, Qinghai | 102.69 | 35.82 | 1932 | Wild | 66-70 |  |  |  | 319.2-531.9 | 9 | -3 | 11/10/10/4/ | 4.5 | 3.102 | 1.194 | 0.4803 | 0.6509 |
| 28 | Lixian, Sichuan | 102.27 | 31.32 | 1880 | Wild | 45-50 |  |  |  | 650-1000 | 11.4 | 2.2 | 12/10/10/10/ | 4.6667 | 3.0221 | 1.2027 | 0.3472 | 0.6546 |
| 29 | Tianshui, Gansu | 106.01 | 34.35 | 1180 | Plantation （afforestation） | 51-55 | ~1969 | 6000 | Hong, He & Zhang 2009 | 491.7 | 11 | -0.4 | 16/10/4/20/ | 5 | 3.2354 | 1.2354 | 0.3722 | 0.6441 |
| 30 | Hanyuan, Sichuan | 102.62 | 29.65 | 1584 | Wild | 38-40 |  |  |  | 600 | 17.8 | 9.9 | 13/10/10/2/ | 5.5 | 3.0022 | 1.2375 | 0.4193 | 0.6253 |
| 31 | Yichang, Hubei | 111.01 | 31.03 | 1432 | Plantation （afforestation） | 65-75 | 1950s-1960s | 666.7 | Xiao et al., 2008 | 1215.6 | 16.9 | 7.3 | 10/10/0/14/ | 4.75 | 3.1851 | 1.1922 | 0.45 | 0.6303 |
| 32 | Ningshan, Shaanxi | 108.54 | 33.55 | 1473 | Plantation （afforestation） | 47-50 |  |  |  | 921 | 12.4 |  | 16/10/1/0/ | 5.25 | 3.1757 | 1.1948 | 0.4062 | 0.6099 |
| 33 | Huxian, Shaanxi | 108.6 | 34.11 | 1677 | Plantation （afforestation） | 58-62 | 1958-1962, 1980 |  | Li 1991 | 627 | 13.5 |  | 20/10/7/10/ | 5.75 | 3.1728 | 1.2373 | 0.4213 | 0.6234 |
| 34 | Liuba, Shaanxi | 106.77 | 33.68 | 1879 | Wild | 40-45 |  |  |  | 886.3 | 11.6 | 0.9 | 9/8/4/10/ | 4.8333 | 3.2366 | 1.1739 | 0.478 | 0.6255 |
| 35 | Kongtong Mountain, Gansu | 106.52 | 35.56 | 1742 | Wild | 60-66 |  |  |  | 537.5 | 10.1 | -2.7 | 5/5/10/10/ | 4.1667 | 3.3107 | 1.2412 | 0.4333 | 0.7329 |
| 36 | Taibai Mountain, Shaanxi | 107.7 | 34.05 | 902 | Wild | 85-90 |  |  |  | 800 | 8.1 | -2.5 | 18/10/3/10/1 | 5.5 | 3.2524 | 1.258 | 0.4779 | 0.646 |
| 37 | Ankang, Shanxi | 109.02 | 32.23 | 714 | Wild | 77-85 |  |  |  | 1050 | 15.7 | 4.8 | 7/7/4/14/ | 4.25 | 2.9236 | 1.1368 | 0.5655 | 0.6379 |
| 38 | Yuexi, Anhui | 116.12 | 31 | 613 | Wild | 48-55 |  |  |  | 1445.8 | 14.4 | 4.3 | 14/10/7/6/ | 5.5 | 3.445 | 1.3391 | 0.5151 | 0.6804 |
| 39 | Zhashui, Shaanxi | 108.9 | 33.82 | 1705 | Wild | 50-58 |  |  |  | 742 | 12.2 | 1.5 | 20/10/5/0/1 | 6.9167 | 3.7292 | 1.4362 | 0.5232 | 0.6806 |
| 40 | Chengxian, Gansu | 105.75 | 33.72 | 966 | Wild | 52-58 |  |  |  | 620.8 | 11.7 | 0.8 | 10/10/1/6/ | 5.1667 | 3.5273 | 1.2998 | 0.475 | 0.6719 |
| 41 | Fengxian, Shaanxi | 106.56 | 33.88 | 964 | Wild | 45-50 |  |  |  | 613 | 12.1 | 1.1 | 12/9/10/2/1 | 4.75 | 2.6789 | 1.1141 | 0.4306 | 0.5833 |
| 42 | Wenxian, Gansu | 104.67 | 32.93 | 935 | Plantation （afforestation） | 35-36 | 1985 |  | Huang 2007 | 400-1000 | 5-15 | 5.3 | 2/2/6/0/ | 2.5833 | 2.3667 | 0.8517 | 0.625 | 0.7083 |
| 43 | CuiHua Mountains, Shaanxi | 109 | 34 | 1036 | Wild | 58-62 |  |  |  | 600 | 15.5 |  | 5/5/2/8/1 | 3.5 | 2.7033 | 0.9872 | 0.4333 | 0.5981 |
| 44 | Ningshan, Shaanxi | 108.8 | 33.83 | 1241 | Wild | 75-80 |  |  |  | 921 | 12.4 | 2.1 | 10/10/3/10/1 | 5.6667 | 3.6169 | 1.3532 | 0.5333 | 0.6816 |
| 45 | Lushi, Henan | 110.95 | 33.15 | 1034 | Wild | 55-60 |  |  |  | 580-680 | 13.8 | 1.7 | 7/7/0/10/ | 4.75 | 3.3736 | 1.2349 | 0.5 | 0.6685 |
| 46 | Lichuan, Hubei | 109.04 | 30.32 | 1077 | Wild | 55-60 |  |  |  | 1200-1400 | 12.3 | 3.9 | 7/7/0/16/ | 4.0833 | 2.9604 | 1.1434 | 0.6012 | 0.666 |
| 47 | Pingliang, Gansu | 106.58 | 35.51 | 1397 | Plantation （afforestation） | 45-50 |  |  | Li 2017 | 537.5 | 10.1 |  | 17/9/5/10/ | 6.1667 | 3.4697 | 1.3465 | 0.5098 | 0.6601 |
| 48 | Hongping, Hubei | 110.45 | 31.68 | 1797 | Wild | 53-55 |  |  |  | 800-2500 | 12.3 |  | 14/10/0/0/ | 6 | 3.8221 | 1.3593 | 0.5298 | 0.6748 |
| 49 | Muzhalin, Henan | 112.25 | 33.78 | 922 | Wild | 48-51 |  |  |  | 830 | 15.2 | 4.4 | 10/8/0/10/ | 4.3333 | 2.9974 | 1.0955 | 0.4833 | 0.6075 |
| 50 | Longquan, Zhejiang | 119.21 | 27.93 | 1144 | Plantation （afforestation） | 60-70 |  |  | Field investigation | 1699.4 | 18 |  | 4/4/0/2/ | 2.75 | 2.2202 | 0.7976 | 0.4375 | 0.5357 |
| 51 | Baojia, Anhui | 116.1 | 30.99 | 1077 | Wild | 58-62 |  |  |  | 1445.8 | 14.4 | 4.3 | 4/4/0/4/ | 3.3333 | 2.7116 | 1.0133 | 0.5833 | 0.6518 |
| 52 | Xinyang, Henan | 114.08 | 31.81 | 678 | Wild | 68-72 |  |  |  | 900-1400 | 15.1-15.3 | 4.7 | 3/3/0/6/ | 2.5833 | 2.2952 | 0.8385 | 0.5 | 0.6222 |
|  | **Planted population** |  |  |  |  |  |  |  |  |  |  |  |  | **4.688** | **3.004** | **1.143** | **0.455** | **0.590** |
|  | **Wild population** |  |  |  |  |  |  |  |  |  |  |  |  | **4.812** | **3.149** | **1.194** | **0.477** | **0.607** |
|  | **QD** |  |  |  |  |  |  |  |  |  |  |  |  | **4.794** | **3.110** | **1.182** | **0.469** | **0.603** |
|  | **All planted population** |  |  |  |  |  |  |  |  |  |  |  |  | **4.621** | **2.885** | **1.096** | **0.419** | **0.563** |
|  | **All wild population** |  |  |  |  |  |  |  |  |  |  |  |  | **4.581** | **2.959** | **1.119** | **0.444** | **0.573** |
|  | **All population** |  |  |  |  |  |  |  |  |  |  |  |  | **4.603** | **2.945** | **1.116** | **0.437** | **0.571** |

*N*, number of individuals; *N*_A_, number of different alleles; *N*_E_, number of effective alleles; *H*_O_ and *H*_E_, observed and expected heterozygosity; *I*, Shannon’s Information Index.

Table S2 PCR primers used in this study.

| Loci | Primer sequence | Repetitive motif | Size/ bp | Ta (℃) | References |
| --- | --- | --- | --- | --- | --- |
| 1_Pc18 (SSR) | F:TTCCCAAAGACCATAGAACCA | (TG)_12_ | 152 | 56 | Salzer, Sebastiani, Gugerli, Buonamici, & Vendramin, 2009 |
|  | R:TCATGAAATATTACGTCCCTTATCC |  |  |  |  |
| 2_RPS1b (SSR) | F: GCCCACTATTCAAGATGTCA | (AC)_10_ | 207 | 59 | Echt, May-Marquardt,  Hseih, & Zahorchak, 1997 |
|  | R:GATGTTAGCAGAAACATGAGG |  |  |  |  |
| 3_RPS90 (SSR) | F:ACCCATTGTGGTGTGTTTGTG | (AC)_23_ | 164 | 56 | Echt, May-Marquardt,  Hseih, & Zahorchak, 1997 |
|  | R:CCTCCGACCATAAACCTTAATG |  |  |  |  |
| 4_RPS119 (SSR) | F:TTGTGAGAAGATACTTCCTCCA | (AC)_10_(AT)_5_ | 205 | 56 | Echt, May-Marquardt,  Hseih, & Zahorchak, 1997 |
|  | R:CCTTGTCTTCTAAAAAACACTTTT |  |  |  |  |
| 5_P37 (SSR) | F: GCTTAGGAGGGAGTCACATA | (AC)_10_ | 154 | 60 | Yu et al., 2012 |
|  | R: CAAGGCAAGACAAGAAATTC |  |  |  |  |
| 6_P5 (SSR) | F: ATTCCTACTTTTCCCGTTT | (CA)_11_ | 120 | 57 | Yu et al., 2012 |
|  | R:ACAGAGACCCCGTTTACAT |  |  |  |  |
| 7_P16 (SSR) | F:GCCAACTACCAACAATCTC | (CA)_14_T(AC)_3_ | 160 | 58 | Yu et al., 2012 |
|  | R:AGTGCCTAAATGTCTTCCA |  |  |  |  |
| 8_P52 (SSR) | F: CCATCCTTCAAATTTTCCT | (AG)_26_ | 138 | 60 | Yu et al., 2012 |
|  | R: GCCATTCTTTCTACCACTT |  |  |  |  |
| 9_P63 (SSR) | F: CTCCTTCTTCATCCATCCATT | (CT)_19_ | 242 | 63 | Yu et al., 2012 |
|  | R: TGAGGTGAGCCTGCATATAGT |  |  |  |  |
| 10_P66 (SSR) | F: GCCACCTCCTAACCCTAAG | (TA)_4_(TG)_3_(T)_3_(GT)_7_ | 159 | 58 | Yu et al., 2012 |
|  | R: ACCCATTTGTCGAATCTAA |  |  |  |  |
| 11_RPtest9 (SSR) | F:CCAGACAACCCAAATGAAGG | (AGC)_10_ | 252 | 61 | Chagné et al., 2004 |
|  | R:GCCTGCTATCGAATCCAGAA |  |  |  |  |
| 12_lw_isotig04306 (SSR) | F: GCCATTTTTTTCTTCTCTCCT | (TCC)_7_ | 190 | 57 | Fang et al., 2014 |
|  | R: GGTCGGTTTCTGAATTTCTAA |  |  |  |  |
| *Ycf*1 (cpDNA) | F: GCCATTTTTTTCTTCTCTCCT |  | 1058 | 54 | Fang et al., 2014 |
|  | R: GGTCGGTTTCTGAATTTCTAA |  |  |  |  |
| nad5 intron1 (mtDNA) | F: AGTCCAATAGGGACAGCACAC |  | 731 | 55 | Jaramillo-Correa et al. 2003 |
|  | R: AGTCCAATAGGGACAGCACAC |  |  |  | Du, Petit, & Liu, 2009 |
| nad7 intron1 (mtDNA) | F: GGAACCGCATATTGGATCAC |  | 681 | 55 | Jaramillo-Correa, Beaulieu  & Bousquet, 2004 |
|  | R: GGTCGGTTTCTGAATTTCTAA |  |  |  |  |
| *AGP*6 (nuDNA) | F: TCAGGGTCAACAATGGCGTTC |  | 441 | 62 |  |
|  | R: GGGCTTTTCAGTGCGGACG |  |  |  |  |
| *LFY* (nuDNA) | F: CTCGTCTATGGTCGTCAT |  | 575 | 58 |  |
|  | R: CAAGCCTTTGTCTGTTCG |  |  |  |  |

SSR, simple sequence repeat; cpDNA, chloroplast DNA fragment; mtDNA, mitochondrial DNA fragments; nuDNA, nuclear DNA fragments.

Table S3 Information concerning the 24 complete chloroplast genomes of *Pinus* used for phylogenomics analysis in the present study.

| *Pinus* | Species | Accession number | | | References |
| --- | --- | --- | --- | --- | --- |
| Subgen. *Pinus* | *P. radiata* | | JN854165 | Parks, Cronn, & Liston, 2012 | |
|  | *P. taeda* | | NC021440 |  | |
|  | *P. ponderosa* | | FJ899555 | Parks, Cronn, & Liston A, 2009 | |
|  | *P. contorta* | | NC011153 | Cronn et al., 2008 | |
|  | *P. merkusii* | | FJ899579 | Parks, Cronn, & Liston A, 2009 | |
|  | *P. roxburghii* | | JN854162 | Parks, Cronn, & Liston, 2012 | |
| Subgen. *Strobus* | *P. monticola* | | FJ899580 | Parks, Cronn, & Liston A, 2009 | |
|  | *P.gerardiana* | | EU998741 | Cronn et al., 2008 | |
|  | *P. krempfii* | | EU998742 | Cronn et al., 2008 | |
|  | *P. longaeva* | | EU998744 | Cronn et al., 2008 | |
|  | *P. nelsonii* | | NC011159 | Cronn et al., 2008 | |
|  | *P. monophylla* | | NC011158 | Cronn et al., 2008 | |
|  | ***P. armandii*** | | **MT583779** | **This study 5_1** | |
|  | ***P. armandii*** | | **MT583780** | **This study 6_1** | |
|  | ***P. armandii*** | | **MT583781** | **This study 14_2** | |
|  | ***P. armandii*** | | **MT583782** | **This study 16_2** | |
|  | ***P. armandii*** | | **MT583783** | **This study 18_2** | |
|  | ***P. armandii*** | | **MT583784** | **This study 19_2** | |
|  | ***P. armandii*** | | **MT583785** | **This study 20_2** | |
|  | ***P. armandii*** | | **MT583786** | **This study 36_3** | |
|  | ***P. armandii*** | | **MT583787** | **This study 39_3** | |
|  | ***P. armandii*** | | **MT644189** | **This study 41_3** | |
|  | ***P. armandii*** | | **MT644190** | **This study 43_3** | |
|  | ***P. armandii*** | | **MT644191** | **This study 44_3** | |

The bold highlight the genomes generated in this study.

Table S4 Prior distributions for model parameters used in divergence model comparisons (Fig. 3a).

| Parameter | Scenario | | |
| --- | --- | --- | --- |
|  | Prior Distribution | Minimum | Maximum |
| Effective population size | | | |
| NA | uniform | 10 | 200000 |
| N1 | uniform | 10000 | 1000000 |
| N2 | uniform | 10000 | 2000000 |
| N3 | uniform | 10000 | 2000000 |
| N4 | uniform | 10000 | 1000000 |
| Time of events | | | |
| t1 | uniform | 10 | 400000 |
| t2 | uniform | 10 | 1000000 |
| Mean mutation rate uniform | uniform | 1.0×10^-7^ | 1.0×10^-5^ |
| Individual locus mutation rate | Gamma | 1.0×10^-8^ | 1.0×10^-5^ |
| Mean coefficient *P* | uniform | 0.1 | 0.7 |
| Individual locus coefficient *P* | Gamma | 0.01 | 0.9 |

Table S5 The seven bioclimatic variables that were used in ecological niche modelling.

| Code | Bioclimatic variable |
| --- | --- |
| BIO2 | Mean Diurnal Range (Mean of monthly (max temp - min temp)) |
| BIO5 | Max Temperature of Warmest Month |
| BIO6 | Min Temperature of Coldest Month |
| BIO7 | Temperature Annual Range (BIO5-BIO6) |
| BIO14 | Precipitation of Driest Month |
| BIO15 | Precipitation Seasonality (Coefficient of Variation) |
| BIO18 | Precipitation of Warmest Quarter |

Table S6 Genetic diversity of cpDNA in the three distinguished lineages of *P. armandii*.

| Lineages | Population | *N* | *H*s | *H*_T_ | *G*_ST_ | *N*_ST_ |
| --- | --- | --- | --- | --- | --- | --- |
| EH | wild | 63 | 0.037 | 0.037 | 0 | 0 |
|  | planted | 28 | 0.185 | 0.333 | 0.444 | 0.444 |
| SH | wild | 107 | 0.133 | 0.131 | -0.015 | 0.004 |
|  | all populations | 135 | 0.144 | 0.170 | 0.150 | 0.174 |
|  | planted | 65 | 0.094 | 0.333 | 0.717 | 0.698 |
| QD | wild | 203 | 0.050 | 0.165 | 0.700 | 0.704 |
|  | all populations | 268 | 0.061 | 0.203 | 0.700 | 0.713 |
|  | planted | 93 | 0.119 | 0.610 | 0.805 | 0.860 |
| all population | wild | 373 | 0.072 | 0.614 | 0.883 | 0.930 |
|  | Total | 466 | 0.081 | 0.612 | 0.868 | 0.915 |

*N*, number of individuals; *N*_A_, number of alleles; *N*_E_, number of effective alleles; *H*_O_ and *H*_E_, observed and expected heterozygosity; *H*_T_, total genetic diversity; *H*_S_, genetic diversity within populations; *G*_ST_, interpopulation differentiation; *N*_ST_, interpopulation differentiation taking similarities between haplotypes into account.

Table S7 Nucleotide variation and haplotype diversity of *P. armandii* across the nuclear loci *AGP*6 and *LFY.*

| Gene locus | Population | Total |  |  |  |  | Nonsynonymous sites | | Silent sites |  | Diversity sites | |  |
| --- | --- | --- | --- | --- | --- | --- | --- | --- | --- | --- | --- | --- | --- |
|  |  | *N* | *L* | *S* | *θ*w | *π*t | *θ*w | *π*a | *θ*w | *π*_sil_ | *N*_h_ | *H*_d_ | *R*m |
|  | planted population | 88 | 441 | 23 | 0.01078 | 0.00962 | 0.00257 | 0.00388 | 0.02997 | 0.02304 | 23 | 0.7375 | 7 |
| *AGP6* | wild population | 354 | 441 | 25 | 0.00880 | 0.00986 | 0.01406 | 0.01208 | 0.00009 | 0.00120 | 79 | 0.8060 | 12 |
|  | all population | 402 | 441 | 26 | 0.00932 | 0.00982 | 0.00293 | 0.00402 | 0.02541 | 0.02272 | 86 | 0.7910 | 12 |
|  | planted population | 12 | 575 | 3 | 0.00381 | 0.00348 | 0.00482 | 0.00440 | 0 | 0 | 3 | 0.7333 | 0 |
| *LFY* | wild population | 76 | 575 | 11 | 0.00425 | 0.00455 | 0.00377 | 0.00428 | 0.00599 | 0.00559 | 14 | 0.8290 | 2 |
|  | all population | 88 | 575 | 11 | 0.00480 | 0.00410 | 0.00557 | 0.00509 | 0.00193 | 0.00038 | 15 | 0.8170 | 2 |

*N*, number of individuals; *L*, length in base pairs; *S*, number of segregating sites; *θ_W_*, Watterson’s parameter (Watterson 1975); *π*_t_, nucleotide diversity across the loci; *π*_a_, nucleotide diversity at nonsynonymous sites; *π*_sil_, nucleotide diversity at silent sites; *N*_h_, number of haplotypes; *H*_d_, haplotype diversity; *R*m, minimum number of recombinant events.

Table S8 Kruskal–Wallis test for genetic diversity differences of microsatellite loci between wild and planted populations of *P. armandii*.

| Lineages | Genetic parameter | Test-Statistic | *P* | FDR_P | Bonferroni_P | Wild_mean | Planted_mean |
| --- | --- | --- | --- | --- | --- | --- | --- |
| SH | *N*_A_ | 0.006 | 0.938 | 0.938 | 1.000 | 4.515 | 4.444 |
|  | *N*_E_ | 1.364 | 0.243 | 0.278 | 1.000 | 2.842 | 2.569 |
|  | *I* | 1.364 | 0.243 | 0.278 | 1.000 | 1.073 | 0.970 |
|  | *H*_O_ | 3.788 | 0.052 | 0.278 | 0.413 | 0.412 | 0.321 |
|  | *H*_E_ | 1.555 | 0.212 | 0.278 | 1.000 | 0.547 | 0.491 |
| QD | *N*_A_ | 0.115 | 0.735 | 0.840 | 1.000 | 4.811 | 4.688 |
|  | *N*_E_ | 0.459 | 0.498 | 0.840 | 1.000 | 3.149 | 3.004 |
|  | *I* | 0.130 | 0.718 | 0.840 | 1.000 | 1.194 | 1.144 |
|  | *H*_O_ | 1.079 | 0.299 | 0.840 | 1.000 | 0.477 | 0.455 |
|  | *H*_E_ | 0.294 | 0.588 | 0.840 | 1.000 | 0.607 | 0.590 |
| all population | *N*_A_ | 0.355 | 0.551 | 0.895 | 1.000 | 4.581 | 4.621 |
|  | *N*_E_ | 0.134 | 0.714 | 0.895 | 1.000 | 2.959 | 2.885 |
|  | *I* | 0.076 | 0.783 | 0.895 | 1.000 | 1.119 | 1.096 |
|  | *H*_O_ | 1.184 | 0.277 | 0.738 | 1.000 | 0.444 | 0.419 |
|  | *H*_E_ | 0.110 | 0.740 | 0.895 | 1.000 | 0.573 | 0.563 |

*N*_A_, number of alleles; *N*_E_, number of effective alleles; *H*_O_ and *H*_E_, observed and expected heterozygosity, respectively; *I*, Shannon’s Information Index.

Table S9 Estimation of rates of contemporary gene flow per generation among three lineages (EH, SH, and QD) using the programs BAYESASS v3.0.

|  | To | | |
| --- | --- | --- | --- |
| From | EH | SH | YP |
| EH | - | 0.024 (0.015) | 0.015(0.010) |
| SH | 0.006 (0.005) | - | 0.021(0.008) |
| YP | 0.004(0.003) | 0.019 (0.006) | - |

EH, East Himalaya; SH, South Hengduan Mountains; QD, Qinling-Daba Mountains. Standard errors are presented in parentheses.

Table S10 Posterior median estimate and 95% highest posterior density interval for demographic parameters of a contraction-expansion model based on the nuclear microsatellite data of the three groups.

| Lineages | Parameters | *N*1 | *N*4 | *N*A | t1 (generations) | t2 (generations) | *μ* | *P* |
| --- | --- | --- | --- | --- | --- | --- | --- | --- |
| EH | Median | 5.78E+05 | 7.11E+04 | 1.32E+05 | 3.68E+03 | 7.17E+03 | 5.30E-06 | 0.416 |
|  | Lower_bound | 1.88E+05 | 1.75E+04 | 7.00E+04 | 5.02E+02 | 2.42E+03 | 2.15E-06 | 0.189 |
|  | Upper_bound | 9.60E+05 | 1.52E+05 | 1.92E+05 | 8.10E+03 | 9.77E+03 | 9.45E-06 | 0.658 |
| SH | Median | 7.39E+05 | 5.83E+04 | 1.31E+05 | 7.34E+03 | 3.92E+05 | 6.58E-06 | 0.488 |
|  | Lower_bound | 3.51E+05 | 1.50E+04 | 6.65E+04 | 2.79E+03 | 4.48E+04 | 3.07E-06 | 0.264 |
|  | Upper_bound | 9.76E+05 | 1.30E+05 | 1.92E+05 | 9.75E+03 | 9.39E+05 | 9.63E-06 | 0.676 |
| QD | Median | 7.71E+05 | 7.67E+04 | 1.56E+05 | 7.58E+03 | 4.61E+05 | 7.22E-06 | 0.432 |
|  | Lower_bound | 4.09E+05 | 1.72E+04 | 9.49E+04 | 3.15E+03 | 7.04E+04 | 3.77E-06 | 0.230 |
|  | Upper_bound | 9.81E+05 | 1.52E+05 | 1.96E+05 | 9.81E+03 | 9.39E+05 | 9.72E-06 | 0.661 |

*N*1, The current population sizes of EH, SH and QD, respectively. *N*A, the ancient population sizes of EH, SH and QD, respectively. *N*4, populations sizes between *N*A and *N*1. t1, time since divergence between *N*1 and *N*4; t2, time since divergence between *N*4 and *N*A; *µ*, mutation rate (per generation per locus).

Table S11 Schoener’s *D* (Schoener, 1968) and Warren’s *I* (Warren et al., 2008) niche overlap statistics and the proportion of niche change among three lineages based on PCA-env niche predictions.

| Lineages | *D* | *I* | Niche unflling | Niche stability | Niche expansion |
| --- | --- | --- | --- | --- | --- |
| EH vs SH | 0.103 | 0.286 | 80.8% | 88.6% | 11.4% |
| EH vs QD | 0.086 | 0.237 | 44.0% | 59.5% | 40.5% |
| SH vs QD | 0.149 | 0.191 | 70.7% | 24.2% | 75.8% |

EH, East Himalaya; SH, South Hengduan Mountains; QD, Qinling-Daba Mountains.


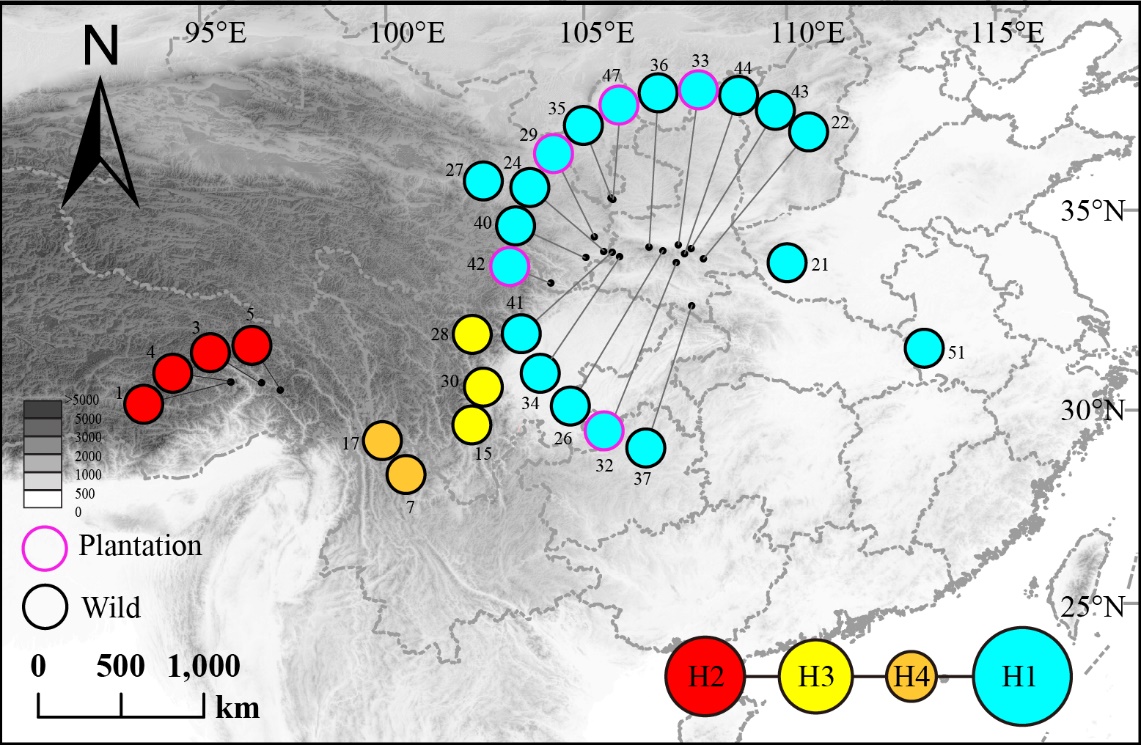


Fig. S1 Geographic distribution and network tree based on haplotypes detected in *P. armandii* using combined data from two mitochondrial fragments (*nad*5 intron 1 and *nad*7 intron 1).


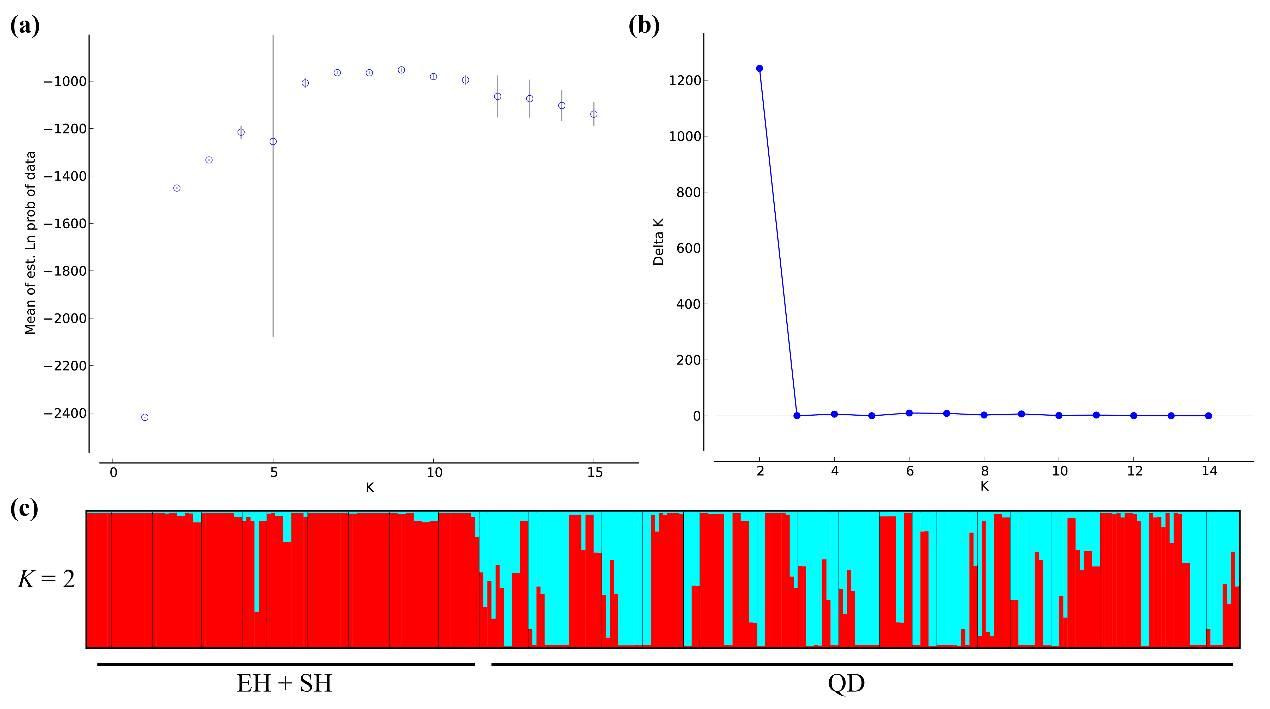


Fig. S2 Bayesian inference analysis of nuclear data for determining the most likely number of clusters (*K*) for *P. armandii*. The distribution of the likelihood L(*K*) values (a) and Delta*K* values (b) are presented for *K* = 1–15 (10 replicates). STRUCTURE plots (c) are presented for the best-performing value, *K* = 2.


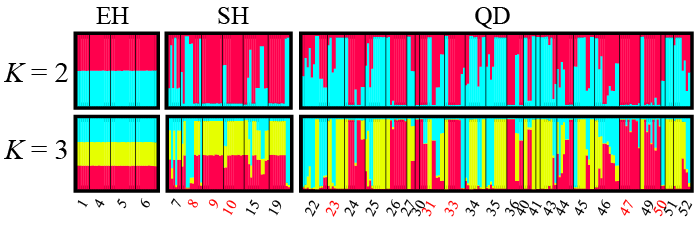


Fig. S3 Bayesian inference analysis of nuclear data for each group separately. STRUCTURE plots are presented for *K* = 2 and *K* = 3. Red numbers denote planted populations.


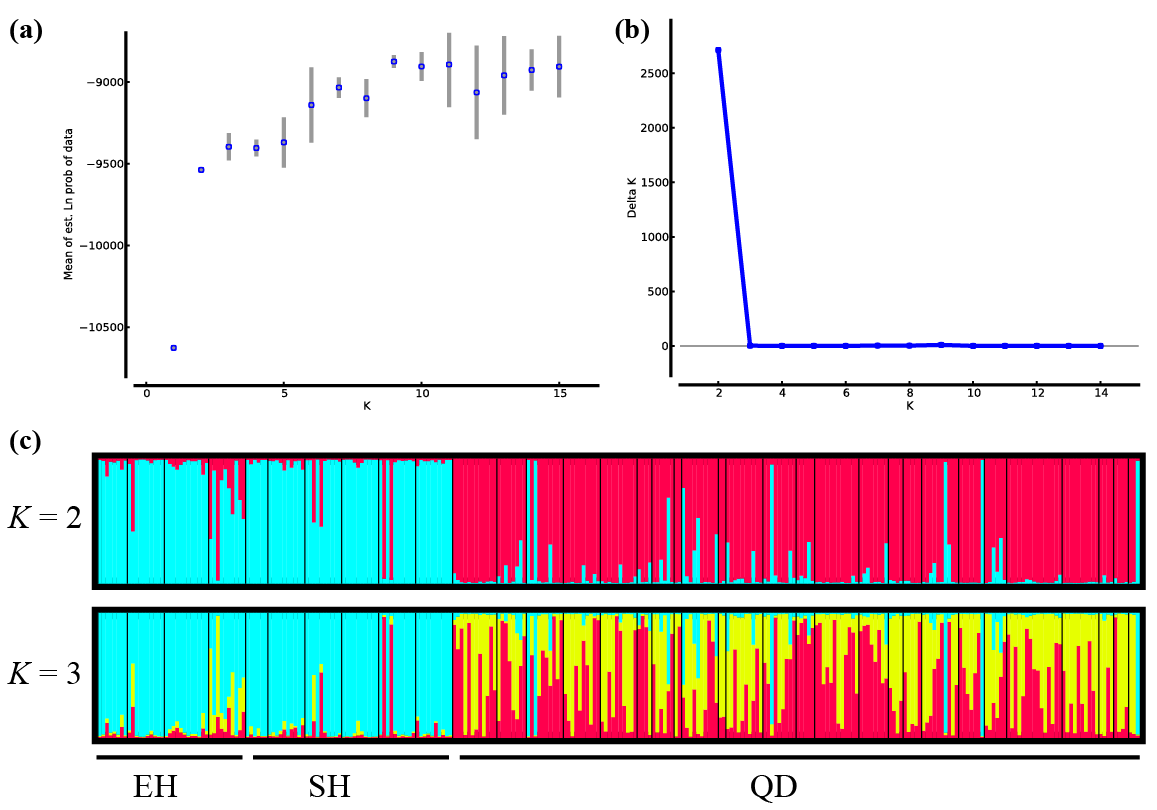


Fig. S4 Bayesian inference analysis of a rarefaction microsatellite dataset which include same number of individuals as nuclear gene dataset for determining the most likely number of clusters (*K*) for *P. armandii*. The distribution of the likelihood L(*K*) values (a) and Delta*K* values (b) are presented for *K* = 1–15 (10 replicates). STRUCTURE plots (c) are presented for the *K* = 2 and *K* = 3.


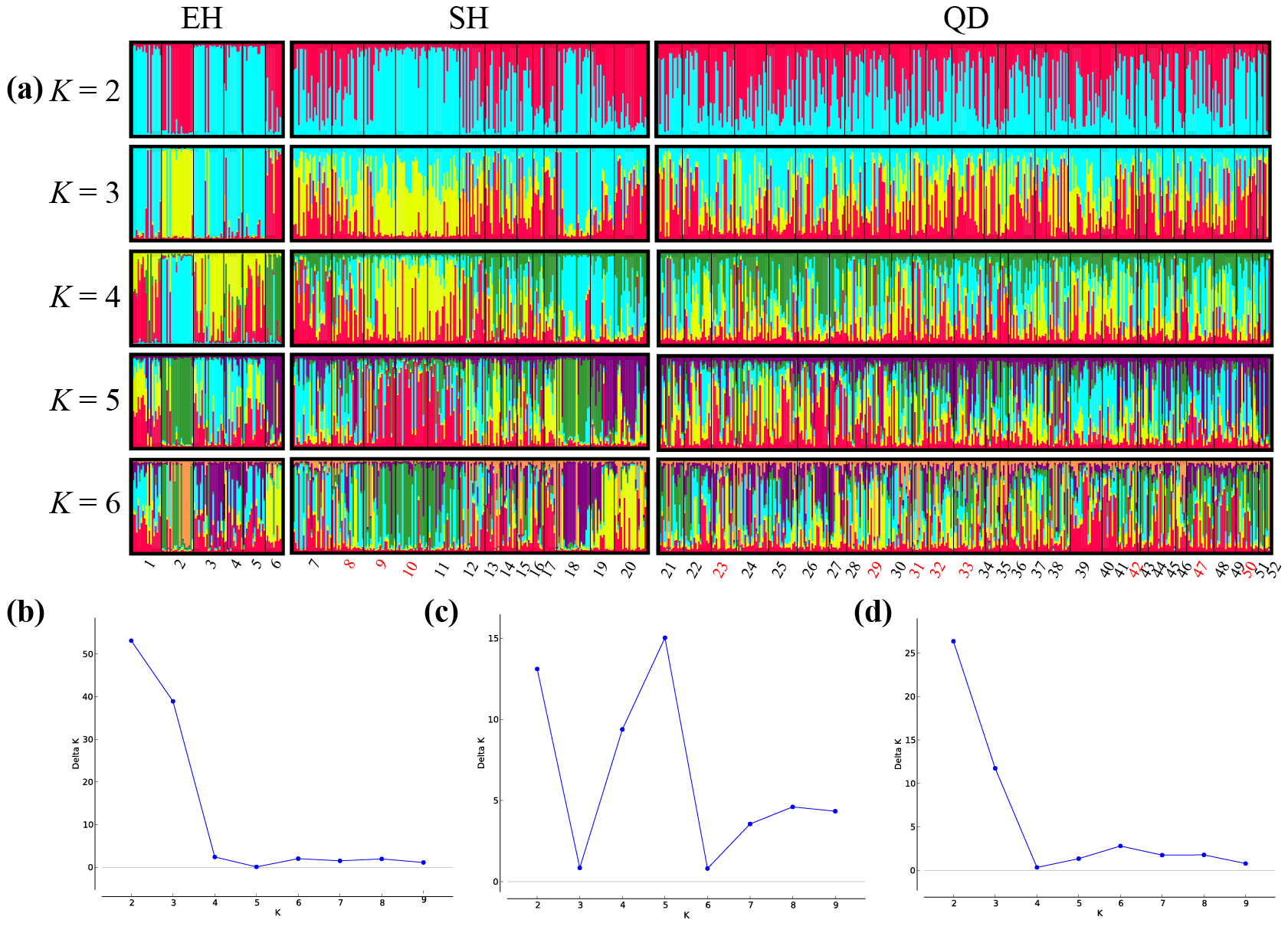


Fig. S5 Bayesian inference analysis of microsatellite data for each group separately. STRUCTURE plots are presented for *K* = 2–6 (a). The distribution of Delta*K* values are presented for *K* = 1–10 (10 replicates) for EH (b), SH (c) and QD (d). Red numbers denote planted populations.


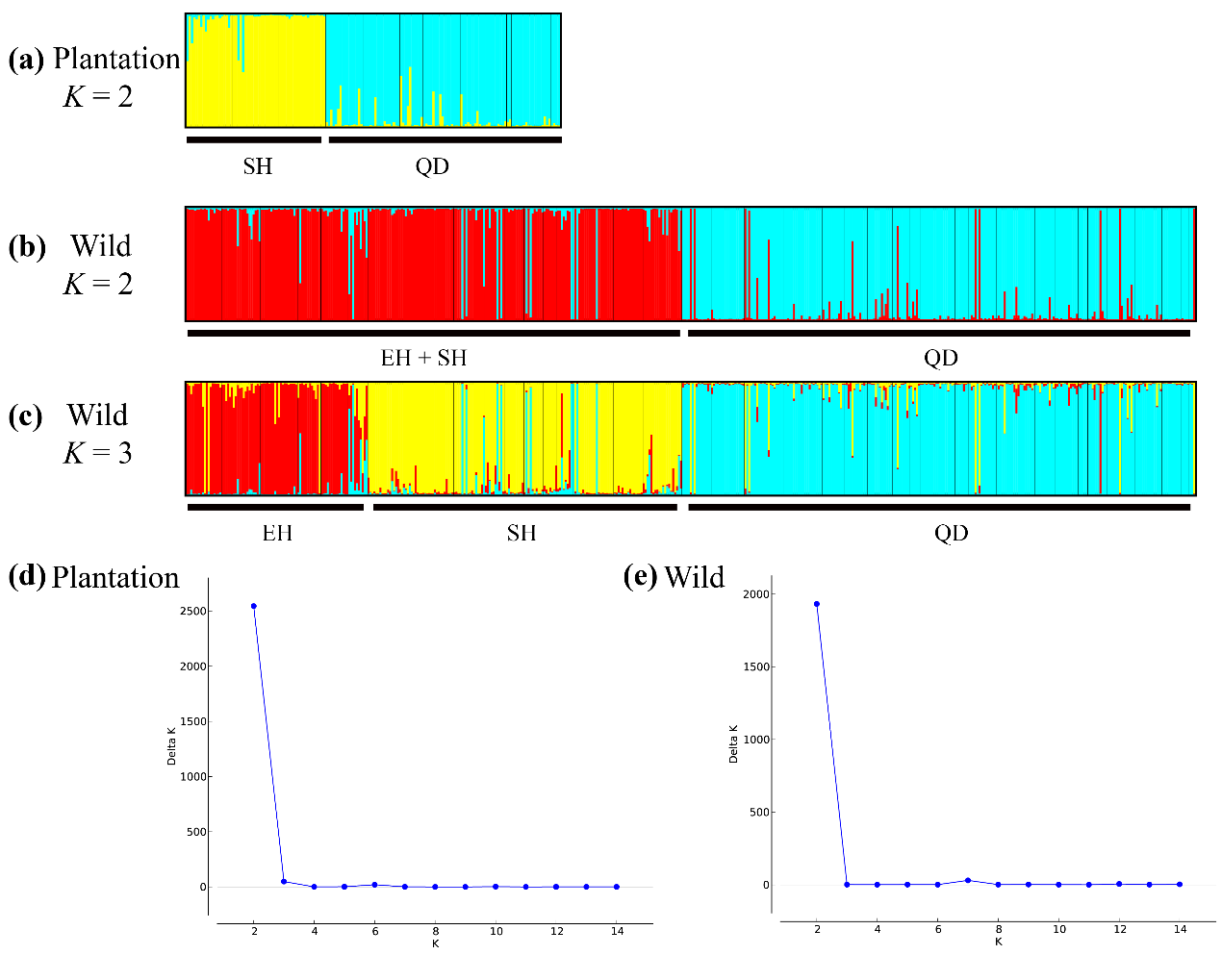


Fig. S6 (a) Genetic structure analyses in planted and wild populations of *P. armandii.* STRUCTURE plots in planted populations of *P. armandii* are presented for the best-performing value, *K* = 2; (b-c) STRUCTURE plots in wild populations of *P. armandii* are presented for *K* = 2 and *K* = 3; (d) The distribution of Delta*K* values are presented for *K* = 1–15 (10 replicates) in planted populations of *P. armandii*; (e) The distribution of Delta*K* values are presented for *K* = 1–15 (10 replicates) in wild populations of *P. armandii*.


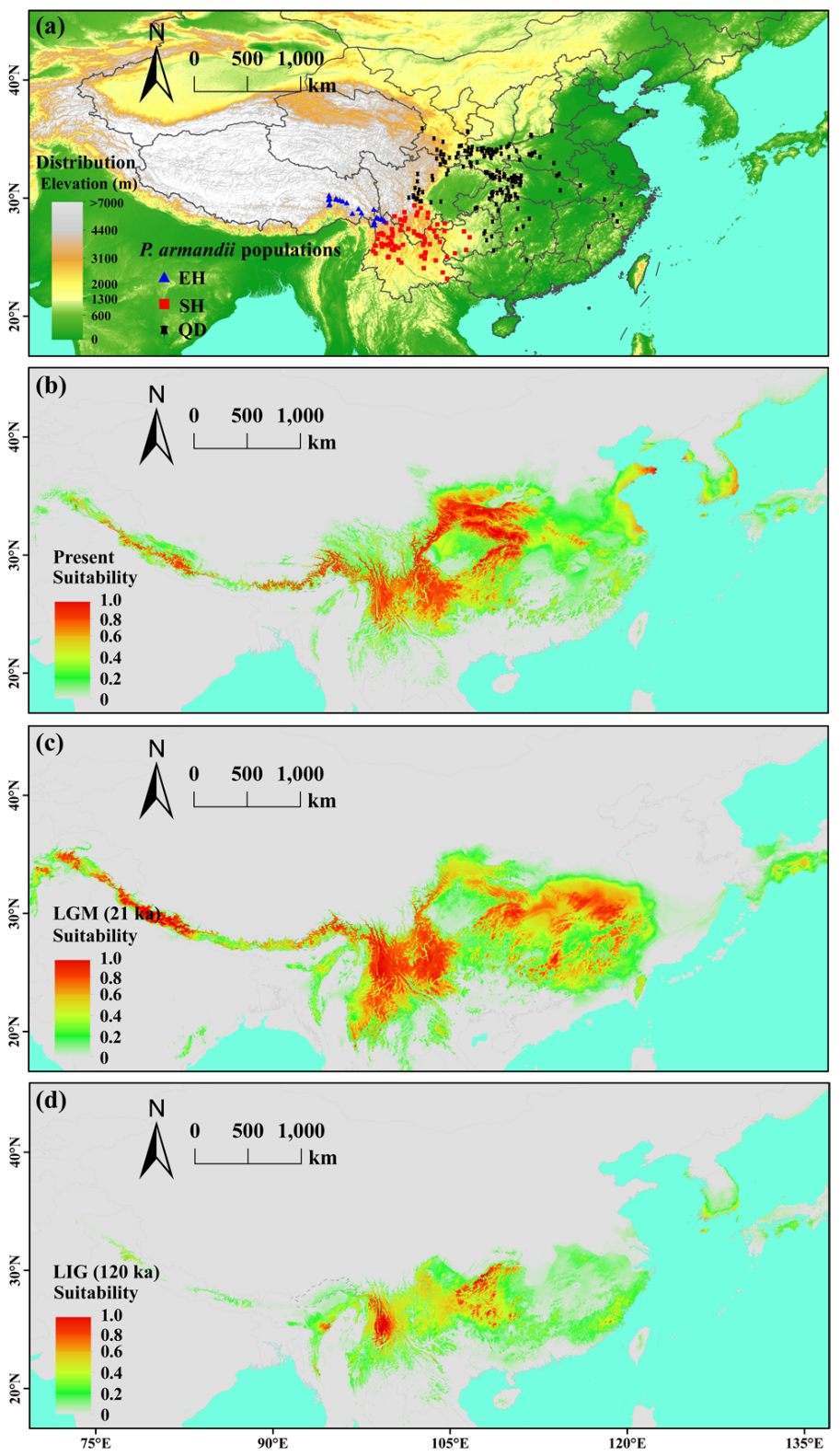


Fig. S7 (a) Map showing a total of 284 distribution records for *P. armandii* sourced from the Global Biodiversity Information Facility (GBIF, <http://www.gbif.org/)>, the National Specimen Information Infrastructure (NSII, <http://www.nsii.org.cn/)>, field work, and literature (Ma, 1989; Liu et al., 2014; Liu et al. 2019). Blue, red and black symbols represent populations of EH, SH and QD respectively. (b-d) Suitable ranges for *P. armandii* during three time periods as predicted by MAXENT: (b) Present, based on conditions *c.* 1950–2000; (c) LGM, last glacial maximum (*c.* 21 000 a BP), and (d) LIG, last interglacial (c. 120 000–140 000 a BP). Red colours indicate higher probabilities of suitable climatic conditions.

**
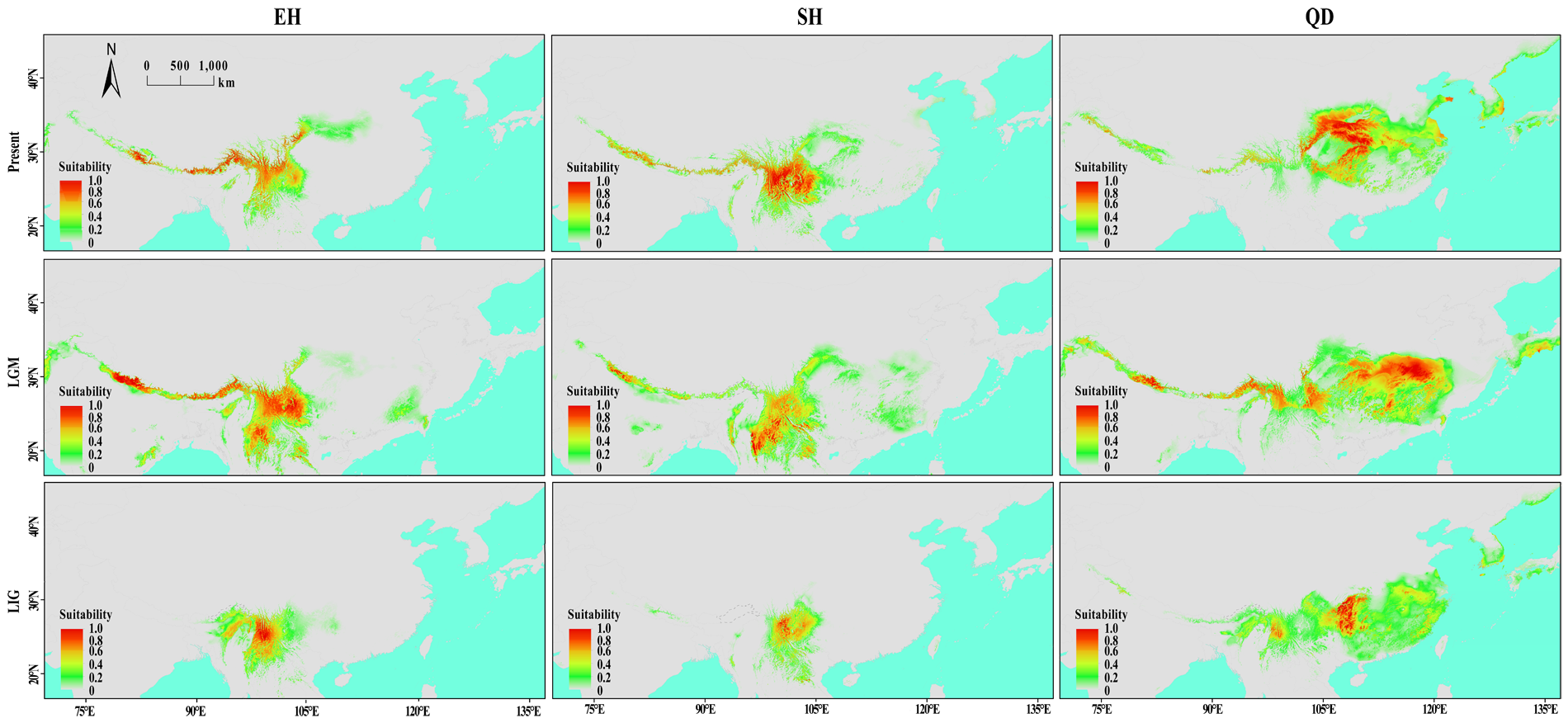
**

Fig. S8 Predicted potential distributions of the three lineages (EH, SH and QD) in *P. armandii* based on ecological niche modelling.

**
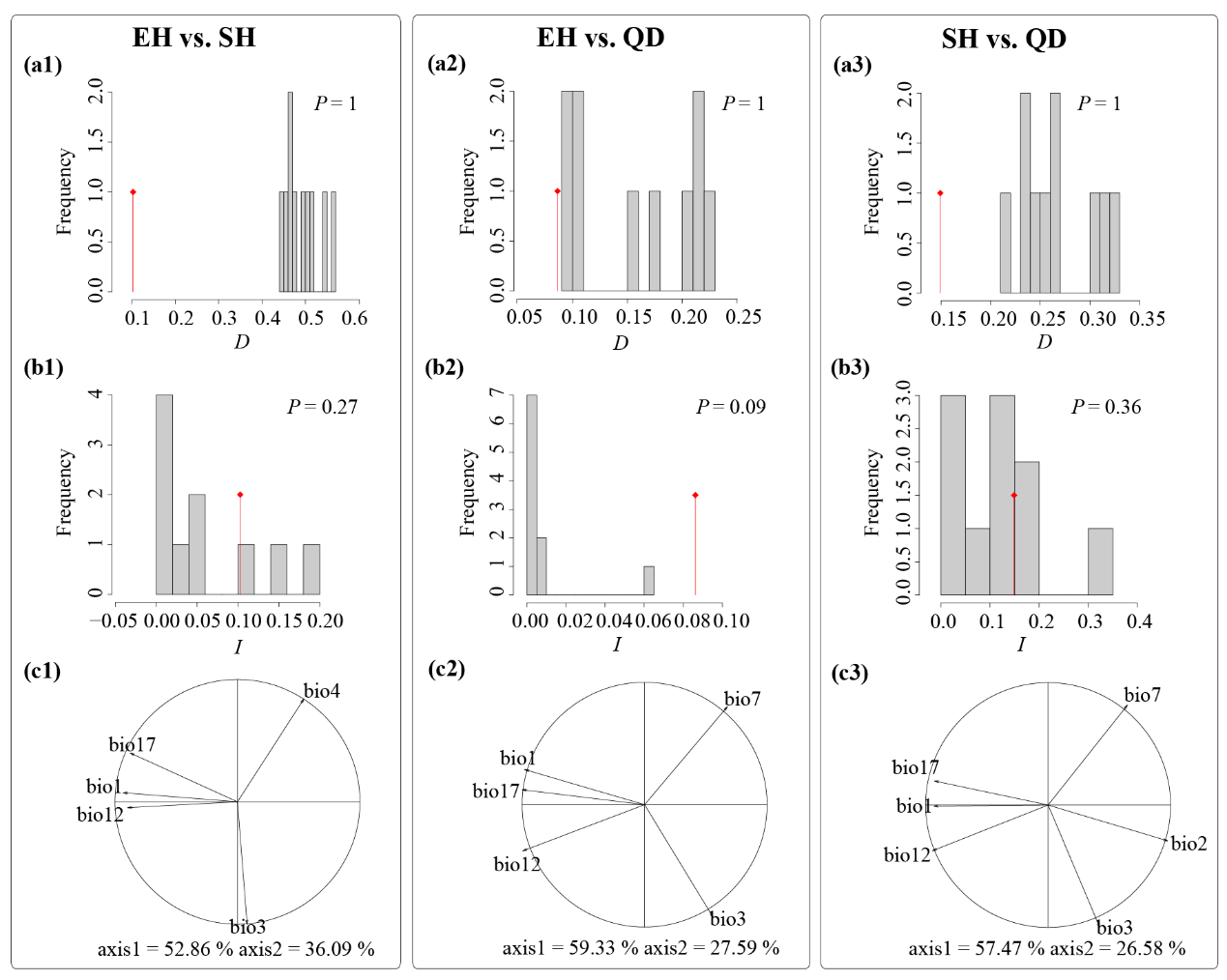
**

Fig. S9 (a1-3) Niche equivalency test for each comparison based on Schoener’s *D* statistic (Schoener 1968) and PCA-env predictions; (b1-3) Niche equivalency test for each comparison based on Warren’s *I* statistic (Warren et al. 2008) and PCA-env predictions; (c1-3) Correlation circle showing the contribution of climatic variables on each axis.

References

Chagné, D., Chaumeil, P., Ramboer, A., Collada, C., Guevara, A., Cervera, M. T., … & Plomion, C. (2004). Cross-species transferability and mapping of genomic and cDNA SSRs in pines. *Theoretical and Applied Genetics*, 109 (6), 1204-1214. doi: 10.1007/s00122-004-1683-z

Cronn, R., Liston, A., Parks, M., Gernandt, D. S., Shen, R., & Mockler, T. (2008). Multiplex sequencing of plant chloroplast genomes using Solexa sequencing-by-synthesis technology. *Nucleic Acids Research*, 36(19), e122. doi: 10.1093/nar/gkn502.

Du, F. K., Petit, R. J., & Liu, J. Q. (2009). More introgression with less gene flow: chloroplast vs. mitochondrial DNA in the Picea asperata complex in China, and comparison with other Conifers. *Molecular Ecology*, 18, 1396-1407. doi: 10.1111/j.1365-294X.2009.04107.x

Echt, C. S., May-Marquardt, P., Hseih, M., & Zahorchak, R. (1997). Characterization of micro-satellite markers in eastern white pine. *Genome*, 39(6), 1102-1108. doi: 10.1139/g97-038

Fang, P., Niu, S., Yuan, H., Li, Z., Zhang, Y., Yuan, L., & Li, W. (2014). Development and characterization of 25 EST-SSR markers in *Pinus sylvestris* var. mongolica (Pinaceae). *Applications in plant sciences*, 2(1), 1300057. doi: 10.3732/apps.1300057

Feng, J., Tang, X. M., & Cui, M. H. (2007). Current situation and management strategy of *Pinus armandii* seed forest in Yema forest farm of Huize. *Journal of Heilongjiang Vocational Institute of Ecological Engineering*, 6, 30–32. doi: CNKI:SUN:HSGX.0.2007-06-016

Hong, Y. J., He, X. A., & Zhang, B. Q. (2009). Effect of thinning on the growth of *Pinus armandii* plantation. *Gansu Science and Technology*, 25, 143–144. doi: CNKI:SUN:GSKJ.0.2009-06-060

Huang, H. L. (2007). Preliminary study on law of height growth of artificial *Pinus armandii*. *Journal of Gansu Forestry Science and Technology*. 32, 52–53. doi: CNKI:SUN:GSLK.0.2007-02-015

Jaramillo-Correa, J. P., Bousquet, J., Beaulieu, J., Isabel, N., Perron, M., & Bouillé M*.* (2003). Cross species amplification of mitochondrial DNA sequencetagged-site markers in conifers: The nature of polymorphism and variation within and among species in *Picea*. *Theoretical and Applied Genetics* , 106, 1353-1367. doi: 10.1007/s00122-002-1174-z

Jaramillo-Correa, J. P., Beaulieu, J., & Bousquet, J. (2004). Variation in mitochondrial DNA reveals multiple distant glacial refugia in black spruce (*Picea mariana*), a transcontinental North American conifer. *Molecular Ecology*, 13, 2735-2747. doi: 10.1111/j.1365-294X.2004.02258.x

Li J. (2017).Cultivation and application of *Pinus armandii* in Taitong Kongtong mountain. *Xiandai Horticulture*, 12, 47. doi: 10.14051/j.cnki.xdyy.2017.24.037

Li, G. C. (1991). Discussion on the methods of forest cutting and regeneration in Taibai forest area. Shaanxi Forest Science and Technology, 1, 31-34.

Liu, L., Hao, Z. Z., Liu, Y. Y., Wei, X. X., Cun, Y. Z., & Wang, X. Q. (2014). Phylogeography of *Pinus armandii* and its relatives: heterogeneous contributions of geography and climate changes to the genetic differentiation and diversification of Chinese white pines. *PLoS One*, 9, e85920. doi:10.1371/journal.pone.0085920

Liu, Y. Y., Jin W. T., Wei, X. X., & Wang, X. Q. (2019). Cryptic speciation in the Chinese white pine (*Pinus armandii*): Implications for the high species diversity of conifers in the Hengduan Mountains, a global biodiversity hotspot. *Molecular Phylogenetics and Evolution*, 138, 114–125. doi: 10.1016/j.ympev.2019.05.015

Parks, M,, Cronn, R., & Liston, A. (2009). Increasing phylogenetic resolution at low taxonomic levels using massively parallel sequencing of chloroplast genomes. *BMC Biology*, 7, 84. doi: 10.1186/1741-7007-7-84

Parks, M., Cronn, R., Liston, A. (2012). Separating the wheat from the chaff: mitigating the effects of noise in a plastome phylogenomic data set from *Pinus* L. (Pinaceae). *BMC Evolutionary Biology*, 12, 100. doi: 10.1186/1471-2148-12-100

Salzer, K., Sebastiani, F., Gugerli, F., Buonamici, A., & Vendramin, G. G. (2009). Isolation and characterization of polymorphic nuclear microsatellite loci in *Pinus cembra* L., 9(3), 858-861. doi: 10.1111/j.1755-0998.2008.02396.x

Schoener, T. W. (1968). The Anolis lizards of Bimini: resource partitioning in a complex fauna. *Ecology*, 49, 704–726.

Warren, D. L., Glor, R. E., & Turelli, M. (2008). Environmental niche equivalency versus conservatism: quantitative approaches to niche evolution. *Evolution*, 62, 2868–2883. doi: 10.1111/j.1558-5646.2008.00482.x.

Xiao, X. F., Qi, W.Y., Wang, J. H., Ying, Z. H., Han, Q. Y., & Li, D. X. (2008). Research on Provenance Selection for *Pinus armandi* Franch. 1, 5–11. doi: CNKI:SUN:FBLI.0.2008-01-002

Yu, J. H., Chen, C. M., Tang, Z. H., Yuan, S. S., Wang, C. J., & Zu, Y. G. (2012). Isolation and characterization of 13 novel polymorphic microsatellite markers for *Pinus koraiensis* (Pinaceae). *American Journal of Botany*, 99(10), e421-424. doi: 10.3732/ajb.1200145
